# Quantitative Proteome Analysis Reveals *Melissa officinalis* Extract Targets Mitochondrial Respiration in Colon Cancer Cells

**DOI:** 10.3390/molecules27144533

**Published:** 2022-07-15

**Authors:** Tzu-Ting Kuo, Li-Chun Lin, Hsin-Yi Chang, Pei-Jung Chiang, Hsin-Yi Wu, Tai-Yuan Chen, Shih-Min Hsia, Tsui-Chin Huang

**Affiliations:** 1 Ph.D. Program for Cancer Molecular Biology and Drug Discovery, College of Medical Science and Technology, Taipei Medical University and Academia Sinica, Taipei 11031, Taiwan; d621107003@tmu.edu.tw (T.-T.K.); d621105001@tmu.edu.tw (L.-C.L.); 2 School of Nutrition and Health Sciences, College of Nutrition, Taipei Medical University, Taipei 11031, Taiwan; bryanhsia@tmu.edu.tw; 3 Graduate Institute of Medical Sciences, National Defense Medical Center, Taipei 11490, Taiwan; hsinyi.chang@mail.ndmctsgh.edu.tw; 4 Department of Research and Development, National Defense Medical Center, Taipei 11490, Taiwan; 5 Graduate School of Pharmaceutical Sciences, Kyoto University, Kyoto 606-8501, Japan; 6 Graduate Institute of Cancer Biology and Drug Discovery, College of Medical Science and Technology, Taipei Medical University, Taipei 11031, Taiwan; b101107008@tmu.edu.tw; 7 Instrumentation Center, National Taiwan University, Taipei 10617, Taiwan; hsinyiwu@ntu.edu.tw; 8 Department of Food Science, National Taiwan Ocean University, Keelung 20224, Taiwan; tychen@mail.ntou.edu.tw; 9 Master Program in Clinical Genomics and Proteomics, College of Pharmacy, Taipei Medical University, Taipei 11031, Taiwan; 10 TMU Research Center of Cancer Translational Medicine, Taipei Medical University, Taipei 11031, Taiwan; 11 Cancer Center, Wan Fang Hospital, Taipei Medical University, Taipei 11031, Taiwan

**Keywords:** *Melissa officinalis*, colorectal cancer, proteomics analysis, reactive oxygen species, mitochondrial respiratory chain complex

## Abstract

*Melissa officinalis* (MO), known as lemon balm, is a popular ingredient blended in herbal tea. In recent decades, the bioactivities of MO have been studied in sub-health and pathological status, highlighting MO possesses multiple pharmacological effects. We previously showed that hot water MO extract exhibited anticancer activity in colorectal cancer (CRC). However, the detailed mechanisms underlying MO-induced cell death remain elusive. To elucidate the anticancer regulation of MO extract in colon cancer, a data-driven analysis by proteomics approaches and bioinformatics analysis was applied. An isobaric tandem mass tags-based quantitative proteome analysis using liquid chromatography–coupled tandem mass spectrometry was performed to acquire proteome-wide expression data. The over-representation analysis and functional class scoring method were implemented to interpret the MO-induced biological regulations. In total, 3465 quantifiable proteoforms were identified from 24,348 peptides, with 67 upregulated and 54 downregulated proteins in the MO-treated group. Mechanistically, MO impeded mitochondrial respiratory electron transport by triggering a reactive oxygen species (ROS)-mediated oxidative stress response. MO hindered the mitochondrial membrane potential by reducing the protein expression in the electron transport chain, specifically the complex I and II, which could be restored by ROS scavenger. The findings comprehensively elucidate how MO hot water extract activates antitumor effects in colorectal cancer (CRC) cells.

## 1. Introduction

*Melissa officinalis* (MO) is a commonly used herbaceous plant belonging to the *Lamiaceae* family. It is also known as lemon balm due to its lemon-like scent. The main constituents known in MO are bioactive: (1) phenolic acids, such as rosmarinic acid, caffeic acid, melitric acid, and sagerinic acid of phenolic acids; (2) flavonoids, such as luteolin and isoquercitrin; (3) triterpenes, including ursolic acid and oleanolic acid; (4) oxygenated monoterpene, such as citronellal and geraniol; (5) sesquiterpene hydrocarbons, including β-cubebene and β-caryophyllene [1,2,3]. The medical use of this herbal plant is proven to have various positive effects on human bodies, such as sedation, neuronal protection, prevention of cardiovascular diseases, pain relief, antiviral, anti-oxidation, and anti-angiogenesis [3,4,5,6,7,8,9]. Previous studies showed that hydroethanolic and aqueous extracts of MO demonstrate promising antitumor potential in CRC cells. Hydroethanolic MO extract inhibited cell proliferation and induced ROS-mediated apoptosis in HT-29 and T84 cells [10]. Our previous study also found that hot water extract of MO exhibited anticancer activity in HCT116 cells by inducing cell cycle arrest, triggering apoptosis, and migration inhibition [11]. However, there are currently no comprehensive studies at the omics scales investigating the mechanism of action of MO extracts on CRC antitumor effects. Owing to numerous bioactive components from MO water extract, it is also hard to discriminate the major action from each ingredient. Comprehensively understanding the mechanism of MO extract by omics-based approaches help us to delineate the complicated regulations.

Quantitative and qualitative proteome analysis has become increasingly popular to investigate complicated biological mechanisms and signal transduction. Several studies applied proteomics approaches to reveal the pharmacological effects, mechanisms of action, and protein targets of natural compounds on cancer treatment [12]. For instance, ethanol extract of *Annona muricata* L. leaves has been found to cause cell apoptosis through the endoplasmic reticulum (ER) stress pathway by using traditional two-dimensional gel-based proteomic analysis in liver cancer [13].

In this study, we performed an isobaric tag-based quantitative proteomics analysis to obtain large-scale proteomics profiling of MO treatment. Isobaric labeling such as isobaric tag for relative and absolute quantitation (iTRAQ) [14] and tandem mass tags (TMT) [15] are widely used in quantitative proteomics due to higher sensitivity, better quantitative accuracy, and allowing different samples performed in one experimental batch [16]. Combining this powerful technique with bioinformatics analysis, we identified participating functions and pathways of MO in CRC cells. We revealed that the primary molecular mechanism of MO in causing HCT116 cell death is dampening the mitochondria function by reactive oxygen species (ROS) production.

## 2. Results

### 2.1. TMT-Based Quantitative Proteomics Analysis of MO Treatment in HCT116 Cells

**Figure 1:**
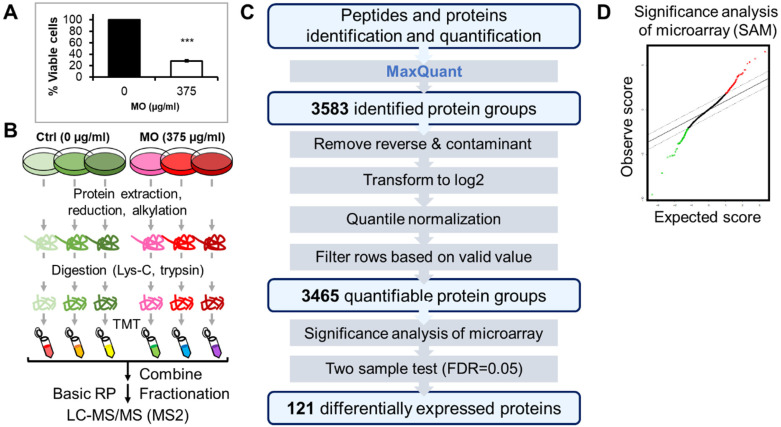
Quantitative proteomics analysis of MO treatment in HCT116 cell line. (**A**) Cell viability of HCT116 cells treated by MO for 48 h. *** *p* < 0.001. (**B**) Schematic representation of the workflow for TMT-based quantitative proteomics. (**C**) The analytical workflow of defining the differentially expressed proteins of MO treatment. (**D**) The quantile-quantile plot from 1000 permutations was plotted from the result of SAM on MO treatment with an FDR of 5%. Red dots indicate upregulated proteins, and green dots indicate downregulated proteins.

To comprehensively explore the antitumor molecular mechanism of MO on CRC cells, we treated cells with MO extract at an effective concentration of 375 µg/mL and performed a TMT-based quantitative proteome analysis on HCT116 cells treated with MO for 48 h (Figure 1A). Three biological replicates from MO-treated and vehicle control were conducted. We labeled the tryptic peptides from each replicate with different isobaric compounds, then fractionated the combined sample into five fractions using Stage-Tip-based basic reversed-phase (RP) material [17] followed by LC/MS/MS analysis (Figure 1B). We used MaxQuant [18] software to identify and quantify peptides and proteins from the mass spectrometry (MS) raw files, and processed the MaxQuant output data for statistical analysis in Persues [19]. For data processing, we removed ‘Potential contaminant’ and ‘Reverse’ hits and transformed the raw intensity into logarithm values. Subsequently, we quantile normalized the log_2_ values of six samples and filtered rows based on valid values with at least two valid values in the Ctrl or the MO group. In total, we identified 3583 protein groups from 24,348 peptides, and 3465 of them were quantifiable (Figure 1C).

**Figure 2:**
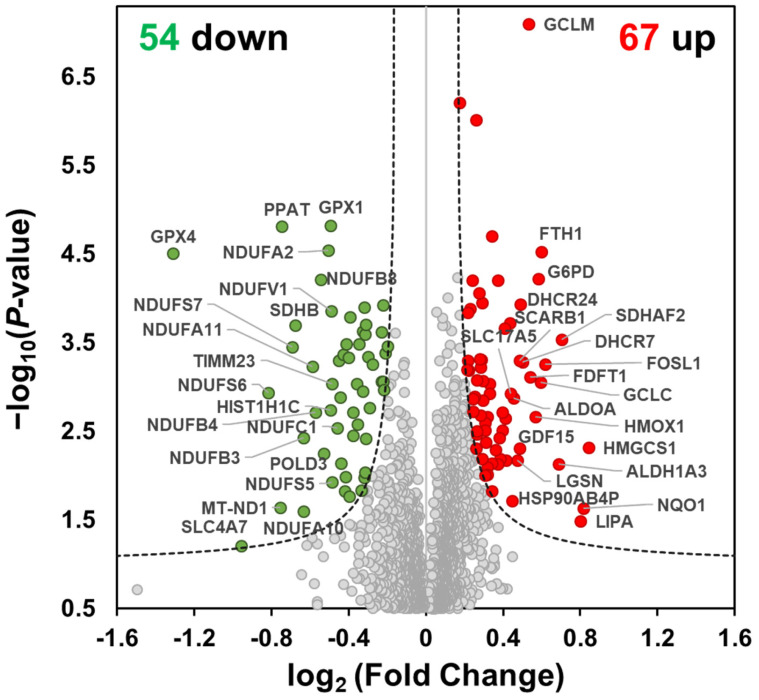
Differentially expressed proteins from the MO-treated HCT116 cells. Volcano plot of MO-treated versus Ctrl-treated proteome in HCT116 cells. *X*-axis represents the protein fold change in log_2,_ and *y*-axis indicates the *p*-value (−log_10_) resulting from a two-sample *t*-test. Upregulated, downregulated, and unchanged proteins were colored red, green, and grey, respectively. The black dashed lines indicate the significance threshold (FDR = 0.05, s0 = 0.07592023 from SAM).

To determine statistically significant proteins, we applied the Significance Analysis of Microarrays (SAM) [20]. SAM is a statistical method designed to identify significant differentially expressed genes from microarray experiments and has been applied to large-scale proteomics data analysis [21]. We calculated the S0 factor from SAM analysis (Figure 1D) and adopted the S0 in a two-sample *t*-test at a false discovery rate (FDR) less than 0.05 in Perseus. As a result, we identified 121 differentially expressed proteins (Supplementary Figure S1) with 54 downregulated and 67 upregulated proteins (Supplementary Tables S1 and S2, respectively). Notably, we found the respiratory complex proteins including NDUFB8, NDUFA2, NDUFS7, NDUFS6, NDUFV1, NDUFA11, NDUFS1, ND1, SDHA, and SDHB were significantly reduced in MO treatment, implying that MO predominantly dysregulates the function of mitochondrial electron transport (Figure 2).

### 2.2. Functional Enrichment Analysis of MO-Regulated Proteins in HCT116 Cells

**Figure 3:**
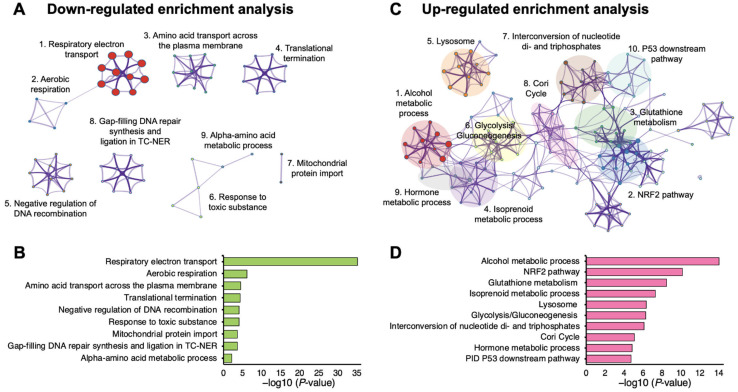
Functional enrichment analysis of differentially expressed proteins of MO treatment compared to control treatment using Metascape. (**A**,**C**) Network of enriched pathways of downregulated (**A**) and upregulated (**C**) proteins of MO treatment compared to control. Each node and its size represent a statistically enriched pathway and the number of enriched proteins on each node pathway, respectively. The clusters of similar pathways are labeled with the same color. (**B**,**D**) Bar charts plot the cluster significance of enriched pathways of downregulated (**B**) and upregulated (**D**) proteins in MO treatment. Each bar showed top one of each enriched cluster pathways. Annotations refer to the gene ontology biological processes (GOBP), Reactome, KEGG, canonical pathways, and WikiPathways. The top pathways ranked by their significance (−log_10_ *p*-value) are shown. The detail information for enriched pathways was listed in Supplementary Tables S3 and S4.

We assumed that MO-disrupted cellular effects are associated with differentially expressed proteins (DEPs) function. To investigate the molecular mechanism regulated by MO in HCT116 cells, we performed pathway enrichment analysis on DEPs using Metascape [22]. Specifically, we used upregulated and downregulated proteins separately as the inputs and searched against databases including GO Biological Processes, Reactome, KEGG, Canonical pathways and WikiPathways. The results showed that the downregulated proteins were significantly enriched in respiratory electron transport, aerobic respiration, amino acid transport across the plasma membrane, translational termination, negative regulation of DNA recombination, and response to a toxic substance (Figure 3A,B). On the other hand, MO-upregulated proteins were enriched in processes such as alcohol metabolic process, NRF2 pathway, and glutathione metabolism (Figure 3C,D). Detailed results of enriched pathways of Metascape analysis are listed in Supplementary Tables S3 and S4 and are consistent with the results from Enrichr analysis (Supplementary Figure S2A–C).

**Figure 4:**
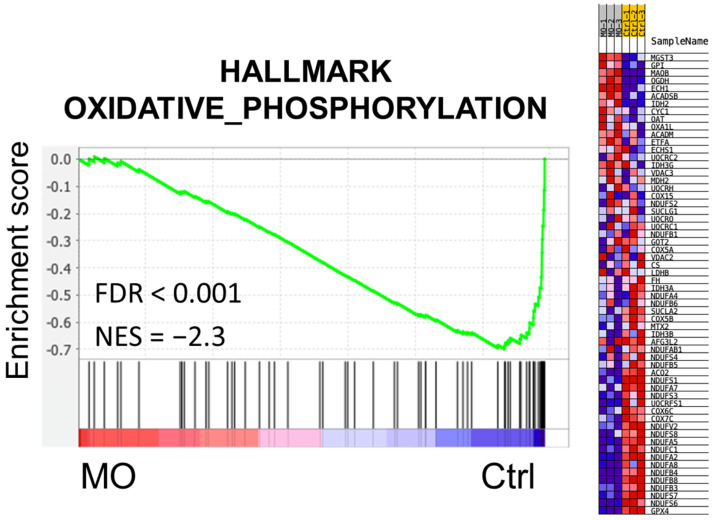
MO diminished oxidative phosphorylation in HCT116 cells. Gene set enrichment analysis (GSEA) was applied to elucidate the effects of MO on CRC cells. Oxidative phosphorylation (OXPHOS) was enriched in the control group with an FDR < 0.001 and NES = −2.3, indicating a strong negative effect of MO on OXPHOS. Proteins involved in OXPHOS are shown on the right panel with their relative expression levels in MO-treated and control groups.

Several methods have been developed for function enrichment analysis. The over-representation analysis (ORA) method directly uses lists of differentially expressed genes or proteins to find significantly enriched terms [23], while the functional class scoring (FCS) method prioritizes the functions based on the expression ranking without prior filters. Gene set enrichment analysis (GSEA), which belongs to the latter, is a ranked-based analysis [24]. Here, we further applied the GSEA to obtain comprehensive effects of MO from a different angle. Consistent with the ORA results, we found that oxidative phosphorylation (OXPHOS) ranked as the top one downregulated pathway under MO treatment (FDR < 0.001, normalized Enrichment Score (NES) = −2.3) (Figure 4). These results indicate that MO might disrupt the redox balance through OXPHOS and induce mitochondria-mediated antitumor effects.

### 2.3. MO Decreased Protein Expression Levels of Mitochondrial Complex I, II, IV

**Figure 5:**
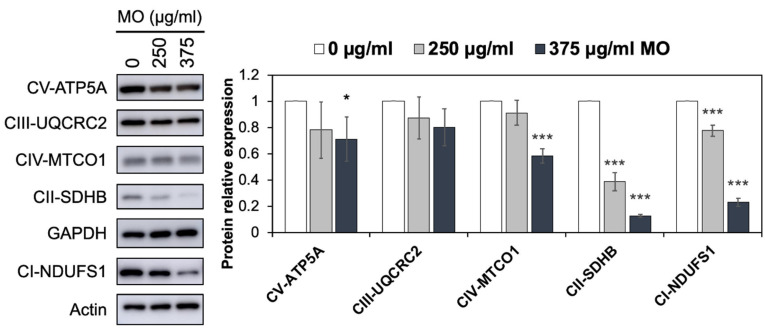
MO decreased the protein expression levels of OXPHOS complex I, II, and IV. HCT116 cells were treated with MO (0, 250, 375 µg/mL) for 48 h. Protein expression levels of OXPHOS were analyzed by western blot and normalized to GAPDH or Actin expression. Bars represent the relative protein expression of complex I-V (CI-V). Relative values (mean ± SD, *n* = 3) were normalized to GAPDH or Actin expression. *** *p* < 0.001, * *p* < 0.05 versus the vehicle control (0 µg/mL MO).

OXPHOS is an essential metabolic process for adenosine triphosphate (ATP) production, which occurs in the inner mitochondrial membrane of eukaryotes and is accompanied by electron transfer. OXPHOS consists of two critical steps, respiratory electron transport and ATP synthesis. The electron transport chain (ETC) comprises complex I (NADH dehydrogenase), complex II (succinate dehydrogenase; SDH), complex III (Ubiquinol–cytochrome c oxidoreductase), and complex IV (cytochrome c oxidase; COX). Complex V is ATP synthase, which utilizes the proton-motive force generated from the ETC to produce ATP [25,26]. In our proteomics and bioinformatics analysis, we found that ETC and OXPHOS functions were notably decreased under MO treatment. 21 out of 54 MO-downregulated proteins are ETC proteins. Among them, 17 belong to complex I, two belong to complex II, and two belong to complex IV (Supplementary Table S5). To evaluate the proteomics result, we measured protein expression levels of complex I to V by western blot with the OXPHOS complexes antibody. Consistent with the proteomics result, we revealed that complex I (NDUFS1), II (SDHB), and IV (MTCO1) were significantly decreased in MO-treated HCT116 cells in a dose-dependent manner (Figure 5). We suggested that inhibiting mitochondrial complexes I, II, and IV by MO might result in mitochondria dysfunction and cause cell death.

### 2.4. MO-Induced Apoptosis and Mitochondrial Dysfunction Were Mediated by ROS Production

**Figure 6:**
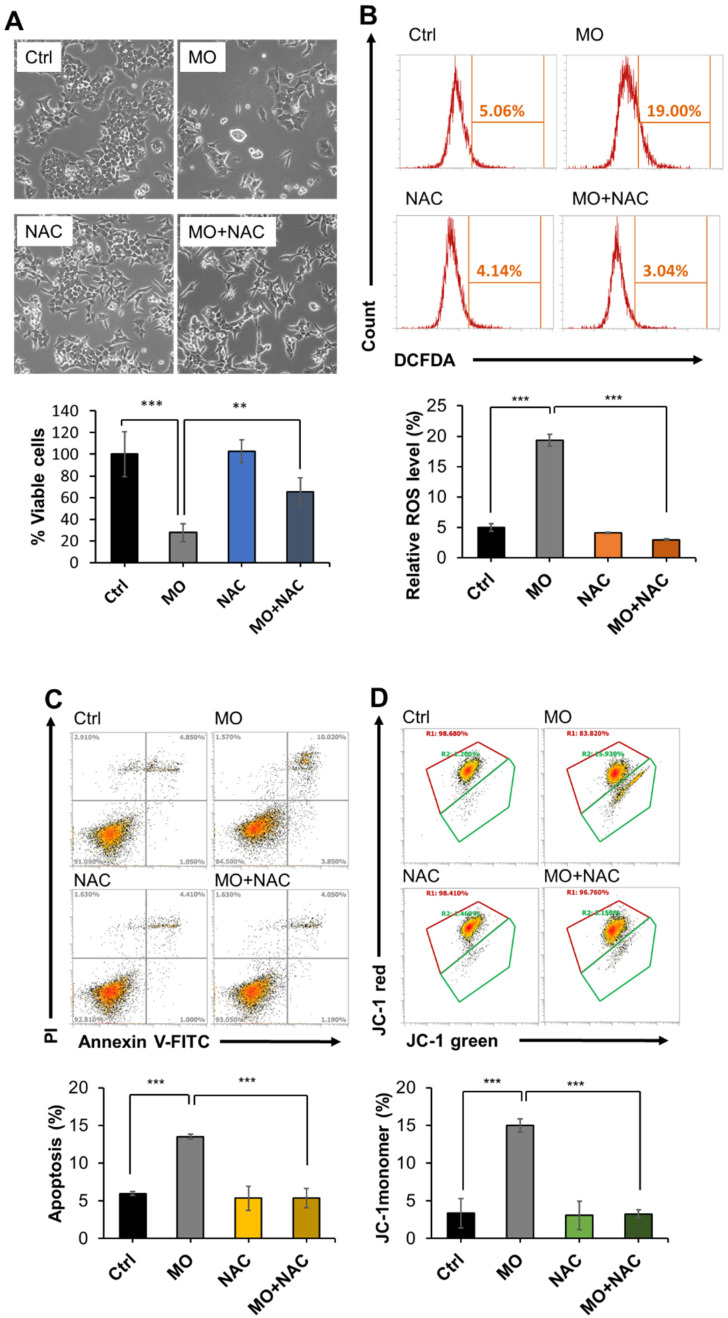

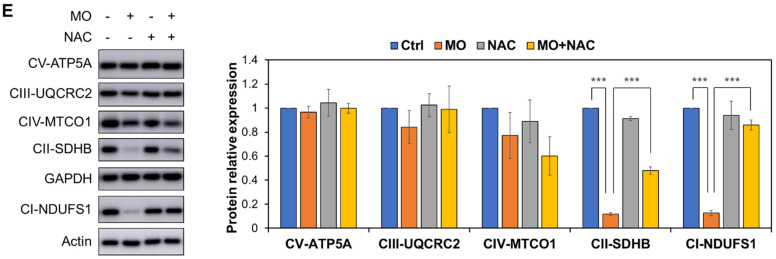
MO-induced apoptosis and mitochondrial dysfunction were mediated by ROS production. HCT116 cells were treated with Ctrl or MO (375 µg/mL) with or without 5 mM NAC for 48 h. After treatment, cell viability was determined by trypan blue exclusion staining (**A**), ROS level was measured by carboxy-H_2_DCFDA staining (**B**), apoptosis analysis was performed based on Annexin V/PI counter stain (**C**), and the MMP was determined by JC-1 red-to-green ratio (**D**). Data from (**B**) to (**D**) were acquired by flow cytometry. All data are presented by the averaged percentage of cell population ± SD from three replicates. (**E**) Protein expression levels of OXPHOS complexes were analyzed by western blot and normalized to GAPDH or Actin expression. Bars represent the relative protein expression of complex I to V (CI to CV). Relative values (mean ± SD, *n* = 3) were normalized to GAPDH or Actin expression and vehicle-treated controls. *** *p* < 0.001, ** *p* < 0.01.

Mitochondria are essential organelles for energy production, cellular metabolism, and cell survival. Mitochondrial membrane potential (MMP), reflecting the process of electron transfer, oxidative phosphorylation, and ATP production, is a critical indicator of mitochondria activity and cell health. Depletion of MMP is one of the mitochondria-mediated apoptosis hallmarks [27]. Combing our previous finding of MO-triggered apoptosis [11] and decreased ETC proteins discovered by the proteomics analysis, we ought to explore whether MO-induced CRC apoptotic cell death is related to mitochondrial dysfunction. To this end, we treated HCT116 cells with 375 µg/mL MO for 48 h and measured MMP using JC-1 dye to assess mitochondria integrity. JC-1 is a membrane-permeant dye which can enter cells and form red fluorescent J-aggregates in the energized mitochondria in healthy cells. However, in an unhealthy state, instead of forming aggregates, JC-1 remains in green fluorescent J-monomer due to the loss of electrochemical potential [28]. We observed that the cells treated with 375 µg/mL MO showed a remarkably lower JC-1 red to green fluorescence ratio, indicating decreased MMP in MO treatment (Figure 6D).

MMP depletion and apoptosis initiation are associated with reactive oxygen species (ROS) levels. ROS is a natural by-product of normal mitochondrial metabolism and participates in cell homeostasis. However, excessive ROS production can be harmful and induce cell death through various mechanisms such as apoptosis [29,30,31,32]. We next investigated whether ROS production is correlated with MO-induced cell death, apoptosis, and mitochondrial dysfunction. We revealed that the ROS level was significantly increased by MO treatment (Figure 6B). Pretreatment with the ROS scavenger N-acetyl-l-cysteine (NAC) significantly lowered ROS level (Figure 6B) and restored MO-caused cytotoxicity (Figure 6A), MO-triggered cell apoptosis (Figure 6C), MMP depletion (Figure 6D), and the expression of OXPHOS complex II and I (Figure 6E). These findings suggest that MO-induced ROS production might serve as a trigger that leads to mitochondrial dysfunction and apoptotic cell death.

## 3. Discussion

As an ancient herbal medicine, *Melissa officinalis* has been widely applied in ethnopharmacology in many countries [3]. In recent decades, many researchers have indicated that MO exhibits multiple pharmacological effects on human cancer cells [9,10,33,34]. Several components in MO, such as rosmarinic acid and luteolin, have been reported to possesses various of biological activities including anticancer effect. Ma et al. revealed that rosmarinic acid significantly induced intracellular ROS production and decreased mitochondrial membrane potential in osteosarcoma cells and led to cell death, and also found RA suppressed cell migration and invasion by inhibiting the expression levels of matrix metalloproteinases and epithelial-mesenchymal transition-associated markers [35]. Luteolin, another component of MO, which has been found to reduce gastric cancer cells growth and cancer metastasis in vitro and in vivo via regulating Notch1, PI3K, AKT, mTOR, ERK, STAT3, and P38 signaling pathways [36].

Previously, we revealed that the hot water MO extract showed anticancer activity in HCT116 CRC cells [11]. Here, we applied proteomic analysis to comprehensively investigate the inhibitory molecular mechanism of MO on CRC cells. For the first time, we revealed that MO-mediated antitumor activity was significantly associated with dysregulation of respiratory electron transport, oxidative phosphorylation pathways, and mitochondrial respiratory chain complex biogenesis (Figure 3 and Figure 4). We identified that 21 proteins from the ETC covering complexes I, II, and IV were diminished by MO, significantly influencing the assembly and biosynthesis of mitochondrial complex I (Supplementary Table S5). In parallel, we also found that MO elevated the Nrf2 pathway and glutathione metabolism, suggesting oxidative stress responses might be triggered. Mechanistically, we confirmed that MO-induced ROS production impairs MMP and leads to apoptotic cell death in HCT116 cells (Figure 6).

Mitochondria play an essential role in our human body due to their functions in energy production and regulation of numerous cellular processes. Mitochondria is a double-layered organelle that harbors unique proteins embedded in the inner and outer membranes. The mitochondrial inner membrane where OXPHOS takes place contains electron transport complexes and ATP synthesis proteins to propagate redox reactions, generate proton gradient, maintain the MMP, and produce ATP [37]. Hence, mitochondria dysfunction and loss of MMP lead to cell death ultimately. Applying proteomics study, we exhibited that MO remarkably decreased the protein expression of mitochondrial respiratory chain components, reflecting the decreased MMP level (Figure 4). Among the downregulated respiratory complexes, complex I is affected by MO the most. The OXPHOS complex I is the biggest ETC complex with the activity to transfer the electron from NADH to coenzyme Q10, coupling protons translocating across the mitochondrial inner membrane. Previous studies showed that inhibiting respiratory complex I is associated with mitochondrial dysfunction and ROS production, resulting in numerous metabolic disorders and apoptotic cancer cell death [38].

Redox homeostasis is critical to maintaining various cellular processes and ensuring cell survival. Regulation of intracellular ROS levels is essential for cell homeostasis. Cancer cells generate higher ROS levels to promote cancer development by affecting cellular metabolism and altering several signaling pathways. However, once the ROS levels exceed the redox capacity of cancer cells, severe oxidative stress occurs and leads to cell death by activating apoptosis, autophagy, ferroptosis, and necroptosis [39]. Many studies have found that natural products and their derivatives possess anticancer properties due to their prooxidant effect on ROS production and result in cancer cells exceeding the tolerable limit of ROS [40]. Previously, we observed that MO extract induced apoptotic cell death in CRC cells [11], which might be related to mitochondrial dysfunction and ROS generation [41]. Weidner et al. also demonstrated that hydroethanolic MO extract induced ROS formation to trigger apoptosis in HT-29 colon cancer cells [10]. In this study, we found that the hot water extract of MO predominantly targeted the ETC complex I to disrupt MMP and induced ROS-mediated apoptosis in HCT116 CRC cells (Figure 6).

From the proteomics results, MO activated the nuclear factor erythroid 2-related factor 2 (NRF2) pathway and glutathione metabolism, suggesting a protective mechanism was induced to prevent oxidative stress. NRF2 is a master transcription factor that accounts for the antioxidant responses. Under normal conditions, NRF2 is targeted by its repressor KEAP1 and E3 ubiquitin ligase Cullin 3 in the cytoplasm, facilitating its ubiquitination and subsequent proteolysis. Upon oxidative stress, NRF2 translocates to the nucleus and regulates several different antioxidant pathways, such as GSH production, regeneration, and utilization [42,43]. Our proteomics revealed that MO significantly induced many NRF2-downstream targets, including GCLC, GCLM, TXN, G6PD, HMOX1, FTH1, and NQO1. On the contrary, MO markedly decreased the protein expression of glutathione peroxidase 1 and 4 (GPX1 and GPX4) (Supplementary Tables S1 and S2). These results demonstrated that cells activate the NRF2 pathway to fight against oxidative stress yet fail to survive ultimately. One of the reasons might be the reduced expression of glutathione peroxidase (GPX), which utilizes GSH as a substrate to reduce ROS [43]. MO increased the levels of the catalytic subunit GCLC and modifier subunit GCLM of the glutamate-cysteine ligase to accelerate the GSH generation flux. However, the synthesized GSH did not meet the redox balance to eliminate ROS due to the uncoupled GPX expression.

Tumor suppressor p53 is the most commonly mutated gene in cancer, including colon cancer. The p53 protein is a transcription factor that responds to cellular stresses, such as DNA damage, oncogene activation, and hypoxia. Activation of p53 leads to cell cycle arrest, senescence, or apoptosis. These biological processes are believed to be the canonical functions of p53 in tumor suppression [44]. In recent years, several studies have highlighted p53 plays a critical role in maintaining the integrity of mitochondria and oxidative phosphorylation and down-regulation of glycolysis [45]. However, the expression of the above-mentioned proteins involved in p53-regulated metabolism was unchanged (HK2, PGAM1, TIGAR, PFKFB3, SCO2, and AIF) or unidentified (PFKFB4) in our proteomics data, inferring p53 only had marginal effects on metabolic regulation in the scenario of MO treatment. The p53 downstream pathway enriched from the proteomics data are specifically the Ferredoxin reductase (FDXR)- and SFN-dependent tumor suppression. FDXR transports electrons from NADPH to mitochondrial cytochrome P450 systems through electron shuttle ferredoxin 1 and converts steroid hormones [46]. FDXR interacts with Fhit to cause electron leakage from ETC, leading to ROS production and apoptosis. As a result, increased FDXR sensitizes cancer cells to oxidative stress-induced apoptosis [47]. SFN encoding 14-3-3 sigma protein is a p53-inducible gene that negatively controls cell cycle progression in response to DNA damage. 14-3-3 sigma causes cell cycle arrest at the G1 phase by inhibiting Cdk2/cyclin E activity and at the G2 phase by sequestering cyclin B/CDC2 to the cytoplasm [48], which is in line with our findings (Figure 6C and [11]). Taken together, the MO-induced p53 activation was mainly involved in activating the oxidative stress associated ROS production and cell death, while to a lesser extent in metabolic regulations.

Ferroptosis is a newly defined programmed cell death that is driven by iron-dependent phospholipid peroxidation and is distinct from apoptosis [49]. Two important regulators were identified in the proteomics result, the GPX4 and HMOX1, indicating MO might induce ferroptosis in addition to apoptosis. The antiporter system x_c_^−^ exchanges intracellular glutamate for extracellular cysteine, followed by intracellular glutathione (GSH) biosynthesis. GSH is a cofactor for GPX4, which converts lipid peroxides to alcohols. Thus, GPX4 inhibition is critical for ferroptosis. Additionally, massive induction of HMOX1 plays another critical regulator in promoting ferroptosis in response to excessive cellular iron and ROS [50]. In parallel, p53 activation also potentiates ferroptosis by inhibiting the transcription of system x_c_^−^ subunit SLC7A11 [51,52], suggesting MO could also activate ferroptosis in line with p53 action. We confirmed that the gene expression SLC7A11 was downregulated and protein expression of p53 and HMOX1 were induced by MO (Supplementary Figure S3A,B). We also verified that the MO-induced HMOX1 overexpression was restored by NAC (Supplementary Figure S3C), suggesting excessive ROS triggers HMOX1-associated regulation in ferroptosis.

Although the current study inspects the effects of MO hot water extract in a comprehensive way, it still exists limitation in terms of identifying the precise contribution from any given ingredient of MO. Also, whether MO extract triggered ferroptosis via lipid peroxidation or iron metabolism remains unclear and is required to be investigated in the future.

## 4. Materials and Methods

### 4.1. Cell Culture

Human colorectal carcinoma cell line HCT116 was purchased from the Bioresource Collection and Research Center (BCRC, Taiwan). The cells were grown in RPMI 1640 medium (Gibco, Grand Island, NY, USA) containing 10% fetal bovine serum (FBS; Gibco) and incubated in a humidified incubator with 5% CO_2_ at 37 °C.

### 4.2. MO Extraction, Chemicals, and Antibodies

MO extract preparation was described in previous studies [11]. In brief, 50 g of the dried MO samples were boiled twice at 100 °C for 30 min in 500 mL of deionized water and clarified by filter papers. The extraction step was repeated twice. All filtrates were combined, freeze-dried to powder, and stored at room temperature. The lyophilized water extract was dissolved in deionized water at 0.5 mg/mL and sterilized by 0.22 μm polyethersulfone membrane before applying to cell culture. Antibodies probing Total OXPHOS Rodent WB Antibody Cocktail was purchased from Abcam (Cambridge, UK). Antibodies probing GAPDH was purchased from Proteintech (Rosemont, IL, USA). Antibodies probing anti-mouse IgG-horseradish peroxidase were purchased from Cell Signaling Technology (Danvers, MA, USA).

### 4.3. Cell Viability Assay

After treatment, cells were trypsinized and counted with trypan blue dye staining (Invitrogen, Carlsbad, CA, USA) to determine the cell viability.

### 4.4. Sample Preparation for Mass Spectrometry (MS) Analysis

HCT116 cells were treated with 375 µg/mL MO water extract or vehicle control for 48 h in triplicates. After treatment, we washed the cells with ice-cold PBS and then lysed them with PTS (phase-transfer surfactant) buffer [53] containing protease and phosphatase inhibitor cocktail (Thermo Fisher Scientific, Waltham, MA, USA). Samples were homogenized for 2 min on ice using homogenizer (UP50H with sonotrode 3; Hielscher, Ultrasound Technology) and clarified by centrifugation at 17,000× *g* for 20 min at 4 °C. Clarified lysate was collected and protein concentration was determined by a BCA Protein Assay (T-Probiotechnology, Taipei, Taiwan). For MS-based analysis, 50 µg of protein from each sample were reduced with 10 mM dithiothreitol (DTT) (Sigma-Aldrich, St. Louis, MO, USA) and alkylated with 50 mM iodoacetamide (Sigma-Aldrich, St. Louis, MO, USA). After alkylation, the proteins were digested with Lys-C endopeptidase (Wako, Tokyo, Japan) (*w*/*w* 1:50) at room temperature for 3 h, followed by trypsin (sequencing grade, Promega Corporation, Madison, WI, USA) (*w*/*w* 1:100) at 37 °C overnight. Trifluoroacetic acid (Wako) and ethyl acetate (EtOAc) (Wako) were added to acidify digested samples and extract detergent, respectively. The resulting peptides were desalted with StageTips [54] packed with SDB-XC Empore disk membranes (GL Sciences, Tokyo, Japan) and quantified by Pierce™ Quantitative Colorimetric Peptide Assay (Thermo Fisher Scientific). An equal amount of desalted peptides from each sample were dried and subjected to TMT labeling.

### 4.5. TMT Labeling and bRP Fractionation

Dried peptides of each sample were reconstituted in 200 mM HEPES and labeled with TMT 10-plex™ Isobaric Label Reagent Set (Thermo) at room temperature for 1 h. Control samples were labeled with TMT reagents 127N, 127C, and 128N, and 375 µg/mL MO-treated samples were labeled with TMT reagents 130N, 130C, and 131, respectively. Labeled samples were quenched with 0.33% hydroxylamine at room temperature for 15 min. All samples were combined, dried, and fractionated using the basic reversed-phase fractionation [17] on SDB-XC StageTips. Resulted fractions were desalted using Pierce^TM^ C18 Spin Tips (Thermo Scientific).

### 4.6. LC-MS/MS Analysis

Five fractionation samples were dried and dissolved in 2% ACN and 0.1% formic acid. Peptides samples were analyzed on Orbitrap Fusion Lumos Tribrid quadrupole-ion trap-Orbitrap mass spectrometer (Thermo Fisher Scientific, San Jose, CA, USA) equipped with an Ultimate system 3000 nanoLC system (Thermo Fisher Scientific, Bremen, Germany). Peptides were loaded into a C18 Acclaim PepMap NanoLC column (25 cm length, 75 µm inner diameter) (Thermo Scientific, San Jose, CA, USA) packed with 2 μm particles with a pore of 100 Å. Mobile phase A was 0.1% formic acid in the water, and mobile phase B was composed of 100% ACN with 0.1% formic acid. Samples were then separated with gradually increasing ACN from 2% to 40% containing 0.1% formic acid in 50 min at a flow rate of 300 nL/min. The mass spectrometer was operated in a data-dependent mode and automatically switched between MS1 and MS2 (MS/MS) acquisition. The MS1 spectra range from 350–1700 *m*/*z* segments were acquired in the Orbitrap at 120,000 resolution at 200 *m*/*z* with AGC target of 5 × 10^5^ and a maximum injection time of 50 ms. The instrument was set to run in top speed mode with 3 s cycles for the survey and the MS/MS scans. Peptide ions with charge states ranging 2 to 7 were sequentially selected with an isolation width of 1.4 Da for fragmentation by higher-energy collisional dissociation (HCD) at normalized collision energy (NCE) of 38%. The resulting fragment spectra were acquired in the Orbitrap mass analyzer with a resolution of 60,000. AGC target of 5 × 10^4^ was set for MS/MS analysis with previously selected ions dynamically excluded for 60 s.

### 4.7. MS Data Processing

The raw MS data files were processed using the MaxQuant [18] software version 1.6.14.0. for peptides and proteins identification and quantification. MS2 data were searched against the Uniprot database of human reference protein (downloaded 12 February 2020) using Andromeda peptide search engine [55]. Reporter ion MS2 was set with corrected 10 plex TMT isobaric labels. Methionine oxidation and N-terminal protein acetylation were set as variable modifications, while carbamidomethylation of cysteine residues was set as fixed modification. Trypsin/P was set as enzyme of specificity digestion mode with max two missed cleavage sites allowed. The matching-between-runs option was enabled with default parameters. After searching, the ProteinGroups output file from MaxQuant was further processed and analyzed with Perseus.

### 4.8. MS Data Analysis and Functional Enrichment Analysis

Data process and statistical analysis were performed in the Perseus software version 1.6.15.0 [56]. After removing decoy hits and contaminations, the log_2_ transformed data were quantile normalized using Normalyzer [57] (http://quantitativeproteomics.org/normalyzer, accessed on 28 May 2021) and then replaced missing values from a normal distribution (down shift: 1.8 s.d., width: 0.3 s.d.). S0 factor was determined using Significance Analysis of Microarrays (SAM) statistical technique with 1000 permutations by samr 3.0 package in R. Two-sample test was performed in Perseus with FDR cut-off of 5% (randomizations = 250, SAM calculated S0 = 0.07592023) to determine statistical significance. Differentially expressed proteins were presented visualized in heatmap and volcano plot. Functional enrichment analysis was performed using Metascape (accessed on 25 June 2021) [22] and gene set enrichment analysis (GSEA) using default setting [24]. For Metascape pathway analysis, GO Biological Processes, Reactome Gene Sets, Kyoto Encyclopedia of Genes and Genomes (KEGG) Pathway, Canonical Pathways and WikiPathways were performed to determine the potential MO-regulated functions with a significance threshold of a minimum count of 3 genes, *p*-value cut-off of 0.01 and minimum enrichment score of 1.5. Gene Ontology (GO) enrichment analysis of Biological Processes (BP); Molecular Functions (MF); Cellular Components (CC) were performed using Enrichr [58] (https://maayanlab.cloud/Enrichr/) (Enrichr Go_BP, MF, CC, accessed on 28 June 2021).

### 4.9. ROS Measurement Assay

For MO and N-acetyl-l-cysteine (NAC) co-treatment, cells were pre-treated with 5 mM NAC for one hour, then were co-treated with MO (375 µg/mL) and NAC for further 48 h. After treatment, cells were harvested and stained with 20 µM carboxy-H_2_DCFDA (Thermo Fisher Scientific) in serum-free RPMI medium at 37 °C for 45 min in the dark. After staining, cells were centrifuged, washed and resuspended in PBS, then the fluorescence level was detected by an Attune NxT acoustic focusing flow cytometer with 488 nm laser for excitation and 530/30 nm filter for detection.

### 4.10. Apoptosis Assay and Mitochondrial Membrane Potential (MMP) Assay

Annexin V/PI staining was performed to detect apoptosis events as described in previous studies [11]. To assess mitochondrial membrane potential (MMP), we harvested MO-treated cells and stained them with JC-1 dye (3 µg/mL in PBS) at 37 °C for 15 min in the dark. The JC-1-stained cells were then analyzed by Attune NxT acoustic focusing flow cytometer (Thermo Fisher Scientific Inc.) with 405 nm laser for excitation. The 512/25 nm and 603/48 nm filters were applied to detect JC-1 monomers (JC-1 green) and J-aggregates (JC-1 red), respectively.

### 4.11. Western Blot Analysis

Cells were treated with MO as indicated concentrations for 48 h. The protein expression level of OXPHOS complexes were determined by western blot following the procedures described previously [11], without heating up the samples. The total OXPHOS human WB antibody cocktail was purchased from Abcam (ab110411). NDUFS1 was purchased from Genetex (GTX113787).

### 4.12. Statistical Analysis

All data were collected from three independent experiments. Data were presented as means ± standard deviations (SD), and the two-tailed Student’s *t*-test was performed to determine the statistically significant difference between MO treated groups and control groups. The *p*-value < 0.05 was considered significant.

## 5. Conclusions

In conclusion, our work elucidates the proteomic molecular mechanism of how MO effectively caused cytotoxicity in CRC cells. We show that the hot water extract of MO preserves the anticancer activity, which boosts oxidative stress and reduces the mitochondrial respiratory capacity to trigger mitochondria-dependent apoptosis in CRC cells.

## Data Availability

Proteomics data have been uploaded into jPOST repository [59] and can be accessed with the identifiers JPST001790 or PXD035338.

## Supplementary Materials

The following are available online at https://www.mdpi.com/article/10.3390/molecules27144533/s1, Table S1: 54 down-regulated proteins in MO treatment by two sample *t*-test. Table S2: 67 up-regulated proteins in MO treatment by two sample *t*-test. Table S3: Nine groups enriched pathways of Metascape analysis of down-regulated proteins in MO treatment. Table S4: Top ten groups enriched pathways of Metascape analysis of up-regulated proteins in MO treatment. Table S5: Down-regulated proteins involved in the mitochondrial respiratory chain complex. Figure S1: Statistical analysis of the MO proteomics results. Figure S2: Gene ontology (GO) enrichment analysis of down-regulated proteins in MO treatment. Figure S3: MO induced ferroptosis-associated molecular regulation.

## Sample Availability

Samples of the compounds are available from the authors.

## Abbreviations:

CRC: colorectal cancer; Ctrl, control; ER, endoplasmic reticulum; ETC, electron transport chain; FDR, false discovery rate; GCLC, glutamate-cysteine ligase catalytic subunit; GCLM, glutamate-cysteine ligase modifier subunit; GO, gene ontology; GO_BP, GO_biological processe; GO_CC, GO_cellular component; GO_MF, GO_molecular function; GPX, glutathione peroxidase; GSEA, gene set enrichment analysis; GSH, glutathione; H_2_O_2_, hydrogen peroxide; KEGG, kyoto encyclopedia of genes and genomes; MMP, mitochondrial membrane potential; MO, *Melissa officinalis*; MS, Mass spectrometry; NER, nucleotide excision repair; NRF2, nuclear factor erythroid 2-related factor 2; OXPHOS, oxidative phosphorylation; ROS, reactive oxygen species; SAM, significance analysis of microarrays; TC-NER, transcription-coupled nucleotide excision repair; TMT, tandem mass tag.
