# Supplementary material for: Quantitative Proteome Analysis Reveals Melissa officinalis Extract Targets Mitochondrial Respiration in Colon Cancer Cells
Source: Molecules. 2022 Jul 15;27(14):4533. doi: 10.3390/molecules27144533 (PMC9316399; doi:10.3390/molecules27144533)
Supplement: Supplementary file 1 [file molecules-27-04533-s001.zip › molecules-1788153-supplementary.pdf]

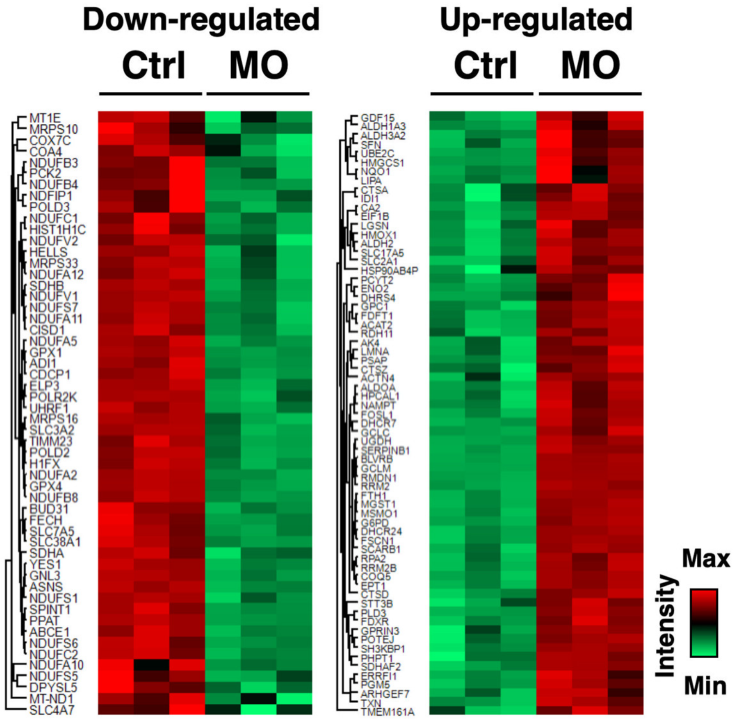

Figure S1. Statistical analysis of the MO proteomics results. Heatmap of up-regulated and down-regulated proteins in MO treatment relative to Ctrl treatment. One column represents one biological replicate under each condition.

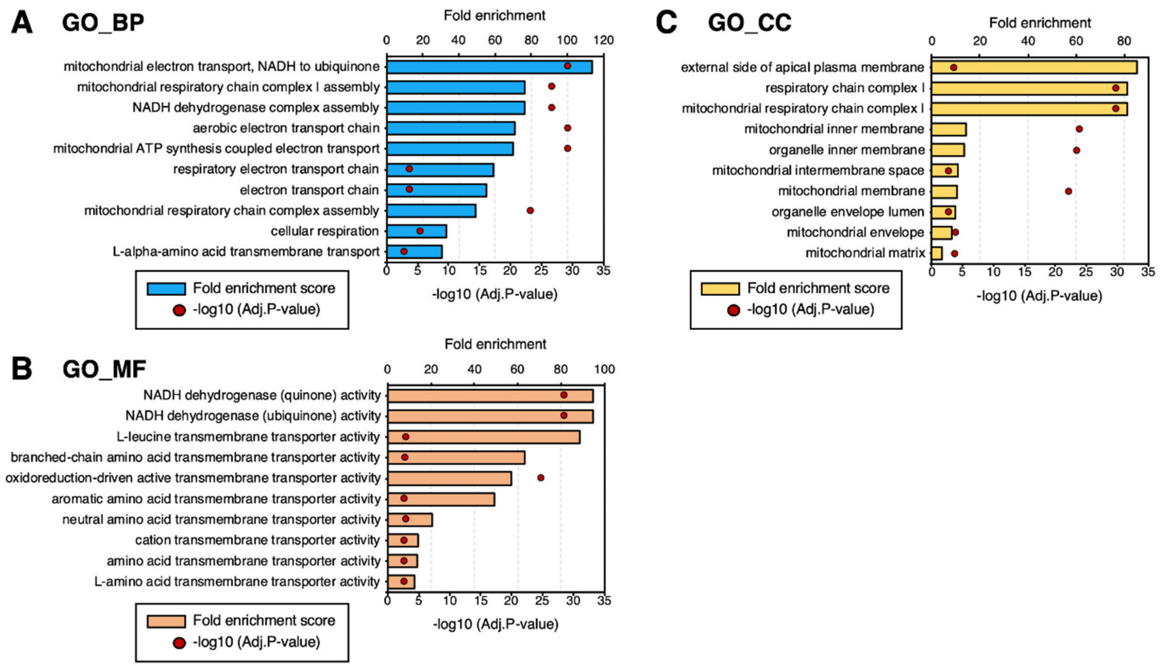

Figure S2. Gene ontology (GO) enrichment analysis of down-regulated proteins in MO treatment. (A,B,C) Top ten of (GO) enrichment analysis of down-regulated proteins in MO-treated HCT116 cells. Categories of GO enrichment analysis included (A) GO_BP: GO Biological Processes (B) GO_MF: GO Molecular Function (C) GO_CC: GO Cellular Component. Fold enrichment were calculated as (number of down-regulated genes in that GO-term/number of down-regulated genes)/(number of total genes in that GO-term/database total genes).

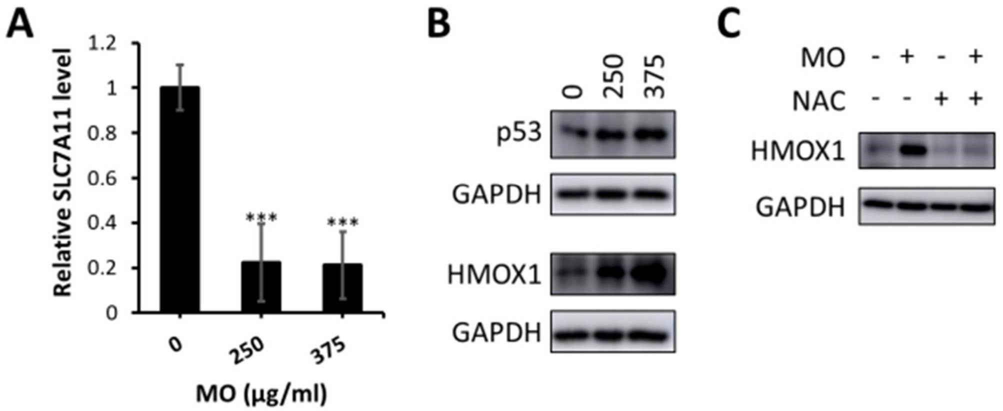

Figure S3. MO induced ferroptosis-associated molecular regulation. (A) The relative mRNA level of SLC7A11. Data were normalized by the expression level of GAPDH. (B) Protein expression of p53 and HMOX1 in CRC cells treated with MO at 0, 250 or 375 μ g/ml. GAPDH was applied as the loading control. (C) Protein expression of HMOX1 in the presence or absence of 375 μ g/ml MO or 5 mM NAC.

Table S1. 54 down-regulated proteins in MO treatment by two sample t-test.

Gene names	Protein IDs	Protein names	Fold change	q-value
GPX4	P36969	Phospholipid hydroperoxide glutathione peroxidase, mitochondrial	0.403	0.00
SLC4A7	Q9Y6M7	Sodium bicarbonate cotransporter 3;Electroneutral sodium bicarbonate exchanger 1	0.515	0.05
NDUFS6	O75380	NADH dehydrogenase [ubiquinone] iron-sulfur protein 6, mitochondrial	0.567	0.01
MT-ND1	P03886	NADH-ubiquinone oxidoreductase chain 1	0.593	0.02
PPAT	Q06203	Amidophosphoribosyltransferase	0.596	0.00
NDUFS7	O75251	NADH dehydrogenase [ubiquinone] iron-sulfur protein 7, mitochondrial	0.618	0.01
SDHB	P21912	Succinate dehydrogenase [ubiquinone] iron-sulfur subunit, mitochondrial	0.625	0.02
NDUFA10	O95299	NADH dehydrogenase [ubiquinone] 1 alpha subcomplex subunit 10, mitochondrial	0.644	0.03
NDUFB3	O43676	NADH dehydrogenase [ubiquinone] 1 beta subcomplex subunit 3	0.644	0.01
NDUFA11	Q86Y39	NADH dehydrogenase [ubiquinone] 1 alpha subcomplex subunit 11	0.666	0.01
NDUFB4	O95168	NADH dehydrogenase [ubiquinone] 1 beta subcomplex subunit 4	0.673	0.01
NDUFB8	O95169	NADH dehydrogenase [ubiquinone] 1 beta subcomplex subunit 8, mitochondrial	0.686	0.01
POLD3	Q15054	DNA polymerase delta subunit 3	0.693	0.02
NDUFA2	O43678	NADH dehydrogenase [ubiquinone] 1 alpha subcomplex subunit 2	0.703	0.01
GPX1	P07203	Glutathione peroxidase 1	0.709	0.02
HIST1H1C	P16403	Histone H1.2	0.710	0.02
NDUFV1	P49821	NADH dehydrogenase [ubiquinone] flavoprotein 1, mitochondrial	0.713	0.01
TIMM23	O14925	Mitochondrial import inner membrane translocase subunit Tim23;Putative mitochondrial import inner membrane translocase subunit Tim23B	0.714	0.01
NDUFS5	O43920	NADH dehydrogenase [ubiquinone] iron-sulfur protein 5	0.714	0.03
NDUFC1	O43677	NADH dehydrogenase [ubiquinone] 1 subunit C1, mitochondrial	0.727	0.02
ABCE1	P61221	ATP-binding cassette sub-family E member 1	0.731	0.01
FECH	P22830	Ferrochelatase, mitochondrial	0.734	0.02
PCK2	Q16822	Phosphoenolpyruvate carboxykinase [GTP], mitochondrial	0.738	0.02

H1FX	Q92522	Histone H1x	0.742	0.01
MRPS10	P82664	28S ribosomal protein S10, mitochondrial	0.746	0.03
NDFIP1	Q9BT67	NEDD4 family-interacting protein 1	0.748	0.03
ADI1	Q9BV57	1,2-dihydroxy-3-keto-5-methylthiopentene dioxygenase	0.751	0.01
POLD2	P49005	DNA polymerase delta subunit 2	0.756	0.01
MT1E	P04732	Metallothionein-1E	0.760	0.04
NDUFA5	Q16718	NADH dehydrogenase [ubiquinone] 1 alpha subcomplex subunit 5	0.761	0.01
NDUFA12	Q9UI09	NADH dehydrogenase [ubiquinone] 1 alpha subcomplex subunit 12	0.770	0.02
SDHA	P31040	Succinate dehydrogenase [ubiquinone] flavoprotein subunit, mitochondrial	0.770	0.02
NDUFC2	O95298	NADH dehydrogenase [ubiquinone] 1 subunit C2	0.781	0.02
HELLS	Q9NRZ9	Lymphoid-specific helicase	0.783	0.03
CDCP1	Q9H5V8	CUB domain-containing protein 1	0.786	0.02
COX7C	P15954	Cytochrome c oxidase subunit 7C, mitochondrial	0.794	0.05
SLC7A5	Q01650	Large neutral amino acids transporter small subunit 1	0.796	0.02
ELP3	Q9H9T3	Elongator complex protein 3	0.797	0.02
COA4	Q9NYJ1	Cytochrome c oxidase assembly factor 4 homolog, mitochondrial	0.801	0.05
ASNS	P08243	Asparagine synthetase [glutamine-hydrolyzing]	0.802	0.02
UHRF1	Q96T88	E3 ubiquitin-protein ligase UHRF1	0.804	0.02
DPYSL5	Q9BPU6	Dihydropyrimidinase-related protein 5	0.804	0.05
NDUFV2	P19404	NADH dehydrogenase [ubiquinone] flavoprotein 2, mitochondrial	0.805	0.03
YES1	P07947	Tyrosine-protein kinase Yes;Proto-oncogene tyrosine-protein kinase Src;Tyrosine-protein kinase Fyn	0.806	0.02
CISD1	Q9NZ45	CDGSH iron-sulfur domain-containing protein 1	0.811	0.02
MRPS33	Q9Y291	28S ribosomal protein S33, mitochondrial	0.816	0.02
SLC38A1	Q9H2H9	Sodium-coupled neutral amino acid transporter 1	0.826	0.02
SLC3A2	P08195	4F2 cell-surface antigen heavy chain	0.853	0.03
NDUFS1	P28331	NADH-ubiquinone oxidoreductase 75 kDa subunit, mitochondrial	0.854	0.04

POLR2K	P53803	DNA-directed RNA polymerases I, II, and III subunit RPABC4	0.856	0.04
GNL3	Q9BVP2	Guanine nucleotide-binding protein-like 3	0.858	0.03
BUD31	P41223	Protein BUD31 homolog	0.860	0.05
MRPS16	Q9Y3D3	28S ribosomal protein S16, mitochondrial	0.864	0.05
SPINT1	O43278	Kunitz-type protease inhibitor 1	0.871	0.05

Table S2. 67 up-regulated proteins in MO treatment by two sample t-test.

Gene names	Protein IDs	Protein names	Fold change	q-value
HMGCS1	Q01581	Hydroxymethylglutaryl-CoA synthase, cytoplasmic	1.793	0.01
NQO1	P15559	NAD(P)H dehydrogenase [quinone] 1	1.760	0.02
LIPA	P38571	Lysosomal acid lipase/cholesteryl ester hydrolase	1.742	0.03
SDHAF2	Q9NX18	Succinate dehydrogenase assembly factor 2, mitochondrial	1.628	0.02
ALDH1A3	P47895	Aldehyde dehydrogenase family 1 member A3	1.610	0.02
FOSL1	P15407	Fos-related antigen 1	1.533	0.01
FTH1	P02794	Ferritin heavy chain;Ferritin heavy chain, N-terminally processed	1.514	0.03
GCLC	P48506	Glutamate--cysteine ligase catalytic subunit	1.510	0.01
G6PD	P11413	Glucose-6-phosphate 1-dehydrogenase	1.497	0.02
HMOX1	P09601	Heme oxygenase 1	1.480	0.01
FDFT1	P37268	Squalene synthase	1.453	0.02
GCLM	P48507	Glutamate--cysteine ligase regulatory subunit	1.447	0.00
DHCR7	Q9UBM7	7-dehydrocholesterol reductase	1.414	0.02
DHCR24	Q15392	Delta(24)-sterol reductase	1.402	0.01
GDF15	Q99988	Growth/differentiation factor 15	1.399	0.02
SCARB1	Q8WTV0	Scavenger receptor class B member 1	1.399	0.02
LGSN	Q5TDP6	Lengsin	1.390	0.02
ALDOA	P04075	Fructose-bisphosphate aldolase A	1.369	0.01
HSP90AB4P	Q58FF6	Putative heat shock protein HSP 90-beta 4	1.364	0.04
SLC17A5	Q9NRA2	Sialin	1.357	0.01
COQ5	Q5HYK3	2-methoxy-6-polyprenyl-1,4-benzoquinol methylase, mitochondrial	1.350	0.02
ARHGEF7	Q14155	Rho guanine nucleotide exchange factor 7	1.334	0.02
AK4	P27144	Adenylate kinase 4, mitochondrial	1.330	0.02
PLD3	Q8IV08	Phospholipase D3	1.327	0.01

HPCAL1	P37235	Hippocalcin-like protein 1	1.316	0.02
POTEJ	P0CG39	POTE ankyrin domain family member J	1.315	0.02
ACTN4	O43707	Alpha-actinin-4	1.307	0.03
ERRFI1	Q9UJM3	ERBB receptor feedback inhibitor 1	1.300	0.02
UGDH	O60701	UDP-glucose 6-dehydrogenase	1.294	0.01
TMEM161A	Q9NX61	Transmembrane protein 161A	1.293	0.03
LMNA	P02545	Prelamin-A/C;Lamin-A/C	1.285	0.02
RRM2	P31350	Ribonucleoside-diphosphate reductase subunit M2	1.266	0.01
SFN	P31947	14-3-3 protein sigma	1.265	0.05
IDI1	Q13907	Isopentenyl-diphosphate Delta-isomerase 1	1.265	0.03
PSAP	P07602	Prosaposin;Saposin-A;Saposin-B-Val;Saposin-B;Saposin-C;Saposin-D	1.258	0.02
TXN	P10599	Thioredoxin	1.256	0.02
UBE2C	O00762	Ubiquitin-conjugating enzyme E2 C	1.248	0.02
CTSA	P10619	Lysosomal protective protein;Lysosomal protective protein 32 kDa chain;Lysosomal protective protein 20 kDa chain	1.248	0.04
ALDH3A2	P51648	Fatty aldehyde dehydrogenase	1.245	0.05
GPRIN3	Q6ZVF9	G protein-regulated inducer of neurite outgrowth 3	1.241	0.03
DHRS4	Q9BTZ2	Dehydrogenase/reductase SDR family member 4	1.237	0.05
NAMPT	P43490	Nicotinamide phosphoribosyltransferase	1.236	0.02
SLC2A1	P11166	Solute carrier family 2, facilitated glucose transporter member 1	1.236	0.03
ACAT2	Q9BWD1	Acetyl-CoA acetyltransferase, cytosolic	1.227	0.02
FDXR	P22570	NADPH:adrenodoxin oxidoreductase, mitochondrial	1.226	0.03
MSMO1	Q15800	Methylsterol monooxygenase 1	1.224	0.02
CTSZ	Q9UBR2	Cathepsin Z	1.222	0.05
CA2	P00918	Carbonic anhydrase 2	1.221	0.02
GPC1	P35052	Glypican-1;Secreted glypican-1	1.218	0.02

PCYT2	Q99447	Ethanolamine-phosphate cytidylyltransferase	1.218	0.03
CTSD	P07339	Cathepsin D;Cathepsin D light chain;Cathepsin D heavy chain	1.213	0.02
SERPINB1	P30740	Leukocyte elastase inhibitor	1.211	0.02
ENO2	P09104	Gamma-enolase	1.202	0.04
PHPT1	Q9NRX4	14 kDa phosphohistidine phosphatase	1.201	0.04
EIF1B	O60739	Eukaryotic translation initiation factor 1b	1.200	0.02
STT3B	Q8TCJ2	Dolichyl-diphosphooligosaccharide--protein glycosyltransferase subunit STT3B	1.196	0.05
BLVRB	P30043	Flavin reductase (NADPH)	1.196	0.02
RPA2	P15927	Replication protein A 32 kDa subunit	1.191	0.03
RDH11	Q8TC12	Retinol dehydrogenase 11	1.186	0.04
PGM5	Q15124	Phosphoglucomutase-like protein 5	1.183	0.04
MGST1	P10620	Microsomal glutathione S-transferase 1	1.182	0.02
FSCN1	Q16658	Fascin	1.170	0.03
SH3KBP1	Q96B97	SH3 domain-containing kinase-binding protein 1	1.165	0.04
ALDH2	P05091	Aldehyde dehydrogenase, mitochondrial	1.163	0.04
EPT1	Q9C0D9	Ethanolaminephosphotransferase 1	1.162	0.03
RRM2B	Q7LG56	Ribonucleoside-diphosphate reductase subunit M2 B	1.158	0.05
RMDN1	Q96DB5	Regulator of microtubule dynamics protein 1	1.128	0.05

Table S3. Nine groups enriched pathways of Metascape analysis of down-regulated proteins in MO treatment. Pathways were enriched from GO Biological Processes, Reactome, KEGG, Canonical pathways and WikiPathways.

Group ID	Category	Term	Description	LogP	Log (q-value)	InTerm _InList	Genes
1	Reactome Gene Sets	R-HSA-611105	Respiratory electron transport	-35.13	-30.83	20/103	COX7C, ND1, NDUFA2, NDUFA5, NDUFA10, NDUFB3, NDUFB4, NDUFB8, NDUFC1, NDUFC2, NDUFS1, NDUFV1, NDUFS5, NDUFS6, NDUFV2, SDHA, SDHB, NDUFA12, NDUFA11, NDUFS7
1	GO Biological Processes	GO:0022904	respiratory electron transport chain	-33.92	-29.92	20/117	COX7C, ND1, NDUFA2, NDUFA5, NDUFA10, NDUFB3, NDUFB4, NDUFB8, NDUFC1, NDUFC2, NDUFS1, NDUFV1, NDUFS5, NDUFS6, NDUFV2, SDHA, SDHB, NDUFA12, NDUFA11, NDUFS7
1	GO Biological Processes	GO:0006120	mitochondrial electron transport, NADH to ubiquinone	-33.60	-29.78	17/55	ND1, NDUFA2, NDUFA5, NDUFA10, NDUFB3, NDUFB4, NDUFB8, NDUFC1, NDUFC2, NDUFS1, NDUFV1, NDUFS5, NDUFS6, NDUFV2, NDUFA12, NDUFA11, NDUFS7
1	Reactome Gene Sets	R-HSA-6799198	Complex I biogenesis	-33.29	-29.66	17/57	ND1, NDUFA2, NDUFA5, NDUFA10, NDUFB3, NDUFB4, NDUFB8, NDUFC1, NDUFC2, NDUFS1, NDUFV1, NDUFS5, NDUFS6, NDUFV2, NDUFA12, NDUFA11, NDUFS7
1	GO Biological Processes	GO:0042775	mitochondrial ATP synthesis coupled electron transport	-33.20	-29.66	19/99	COX7C, ND1, NDUFA2, NDUFA5, NDUFA10, NDUFB3, NDUFB4, NDUFB8, NDUFC1, NDUFC2, NDUFS1, NDUFV1, NDUFS5, NDUFS6, NDUFV2, SDHA, NDUFA12,

							NDUFA11, NDUFS7
1	Reactome Gene Sets	R-HSA-163200	Respiratory electron transport, ATP synthesis by chemiosmotic coupling, and heat production by uncoupling proteins.	-33.15	-29.66	20/127	COX7C, ND1, NDUFA2, NDUFA5, NDUFA10, NDUFB3, NDUFB4, NDUFB8, NDUFC1, NDUFC2, NDUFS1, NDUFV1, NDUFS5, NDUFS6, NDUFV2, SDHA, SDHB, NDUFA12, NDUFA11, NDUFS7
1	GO Biological Processes	GO:0042773	ATP synthesis coupled electron transport	-33.11	-29.66	19/100	COX7C, ND1, NDUFA2, NDUFA5, NDUFA10, NDUFB3, NDUFB4, NDUFB8, NDUFC1, NDUFC2, NDUFS1, NDUFV1, NDUFS5, NDUFS6, NDUFV2, SDHA, NDUFA12, NDUFA11, NDUFS7
1	KEGG Pathway	hsa00190	Oxidative phosphorylation	-32.72	-29.37	20/133	COX7C, ND1, NDUFA2, NDUFA5, NDUFA10, NDUFB3, NDUFB4, NDUFB8, NDUFC1, NDUFC2, NDUFS1, NDUFV1, NDUFS5, NDUFS6, NDUFV2, SDHA, SDHB, NDUFA12, NDUFA11, NDUFS7
1	KEGG Pathway	ko00190	Oxidative phosphorylation	-32.72	-29.37	20/133	COX7C, ND1, NDUFA2, NDUFA5, NDUFA10, NDUFB3, NDUFB4, NDUFB8, NDUFC1, NDUFC2, NDUFS1, NDUFV1, NDUFS5, NDUFS6, NDUFV2, SDHA, SDHB, NDUFA12, NDUFA11, NDUFS7
1	WikiPathways	WP111	Electron Transport Chain (OXPHOS system in mitochondria)	-32.59	-29.29	19/106	COX7C, ND1, NDUFA2, NDUFA5, NDUFA10, NDUFB3, NDUFB4, NDUFB8, NDUFC1, NDUFC2, NDUFS1, NDUFV1, NDUFS5, NDUFS6, NDUFV2, SDHA, SDHB, NDUFA12, NDUFS7

1	GO Biological Processes	GO:0010257	NADH dehydrogenase complex assembly	-32.17	-28.95	17/65	ND1, NDUFA2, NDUFA5, NDUFA10, NDUFB3, NDUFB4, NDUFB8, NDUFC1, NDUFC2, NDUFS1, NDUFV1, NDUFS5, NDUFS6, NDUFV2, NDUFA12, NDUFA11, NDUFS7
1	GO Biological Processes	GO:0032981	mitochondrial respiratory chain complex I assembly	-32.17	-28.95	17/65	ND1, NDUFA2, NDUFA5, NDUFA10, NDUFB3, NDUFB4, NDUFB8, NDUFC1, NDUFC2, NDUFS1, NDUFV1, NDUFS5, NDUFS6, NDUFV2, NDUFA12, NDUFA11, NDUFS7
1	KEGG Pathway	hsa05012	Parkinson's disease	-32.11	-28.92	20/142	COX7C, ND1, NDUFA2, NDUFA5, NDUFA10, NDUFB3, NDUFB4, NDUFB8, NDUFC1, NDUFC2, NDUFS1, NDUFV1, NDUFS5, NDUFS6, NDUFV2, SDHA, SDHB, NDUFA12, NDUFA11, NDUFS7
1	GO Biological Processes	GO:0045333	cellular respiration	-31.60	-28.45	21/187	COX7C, ND1, NDUFA2, NDUFA5, NDUFA10, NDUFB3, NDUFB4, NDUFB8, NDUFC1, NDUFC2, NDUFS1, NDUFV1, NDUFS5, NDUFS6, NDUFV2, SDHA, SDHB, CISD1, NDUFA12, NDUFA11, NDUFS7
1	KEGG Pathway	hsa05016	Huntington's disease	-31.30	-28.21	21/193	COX7C, GPX1, NDUFA2, NDUFA5, NDUFA10, NDUFB3, NDUFB4, NDUFB8, NDUFC1, NDUFC2, NDUFS1, NDUFV1, NDUFS5, NDUFS6, NDUFV2, POLR2K, SDHA, SDHB, NDUFA12, NDUFA11, NDUFS7
1	KEGG Pathway	ko05016	Huntington's disease	-31.30	-28.21	21/193	COX7C, GPX1, NDUFA2, NDUFA5, NDUFA10, NDUFB3, NDUFB4, NDUFB8, NDUFC1, NDUFC2, NDUFS1, NDUFV1, NDUFS5,

							NDUFS6, NDUFV2, POLR2K, SDHA, SDHB, NDUFA12, NDUFA11, NDUFS7
1	GO Biological Processes	GO:0033108	mitochondrial respiratory chain complex assembly	-30.77	-27.70	18/101	ND1, NDUFA2, NDUFA5, NDUFA10, NDUFB3, NDUFB4, NDUFB8, NDUFC1, NDUFC2, NDUFS1, NDUFV1, NDUFS5, NDUFS6, NDUFV2, COA4, NDUFA12, NDUFA11, NDUFS7
1	GO Biological Processes	GO:0022900	electron transport chain	-30.04	-27.02	20/178	COX7C, ND1, NDUFA2, NDUFA5, NDUFA10, NDUFB3, NDUFB4, NDUFB8, NDUFC1, NDUFC2, NDUFS1, NDUFV1, NDUFS5, NDUFS6, NDUFV2, SDHA, SDHB, NDUFA12, NDUFA11, NDUFS7
1	Reactome Gene Sets	R-HSA-1428517	The citric acid (TCA) cycle and respiratory electron transport	-30.04	-27.02	20/178	COX7C, ND1, NDUFA2, NDUFA5, NDUFA10, NDUFB3, NDUFB4, NDUFB8, NDUFC1, NDUFC2, NDUFS1, NDUFV1, NDUFS5, NDUFS6, NDUFV2, SDHA, SDHB, NDUFA12, NDUFA11, NDUFS7
1	GO Biological Processes	GO:0006119	oxidative phosphorylation	-29.63	-26.64	19/148	COX7C, ND1, NDUFA2, NDUFA5, NDUFA10, NDUFB3, NDUFB4, NDUFB8, NDUFC1, NDUFC2, NDUFS1, NDUFV1, NDUFS5, NDUFS6, NDUFV2, SDHA, NDUFA12, NDUFA11, NDUFS7
1	KEGG Pathway	ko04932	Non-alcoholic fatty liver disease (NAFLD)	-29.57	-26.60	19/149	COX7C, NDUFA2, NDUFA5, NDUFA10, NDUFB3, NDUFB4, NDUFB8, NDUFC1, NDUFC2, NDUFS1, NDUFV1, NDUFS5, NDUFS6, NDUFV2, SDHA, SDHB, NDUFA12,

							NDUFA11, NDUFS7
1	WikiPathways	WP4396	Nonalcoholic fatty liver disease	-28.95	-26.00	19/160	COX7C, NDUFA2, NDUFA5, NDUFA10, NDUFB3, NDUFB4, NDUFB8, NDUFC1, NDUFC2, NDUFS1, NDUFV1, NDUFS5, NDUFS6, NDUFV2, SDHA, SDHB, NDUFA12, NDUFA11, NDUFS7
1	WikiPathways	WP4324	Mitochondrial complex I assembly model OXPHOS system	-28.50	-25.57	15/56	ND1, NDUFA2, NDUFA5, NDUFA10, NDUFB3, NDUFB4, NDUFB8, NDUFC1, NDUFC2, NDUFS1, NDUFV1, NDUFS5, NDUFS6, NDUFV2, NDUFA12
1	KEGG Pathway	hsa04932	Non-alcoholic fatty liver disease (NAFLD)	-28.48	-25.56	19/169	COX7C, NDUFA2, NDUFA5, NDUFA10, NDUFB3, NDUFB4, NDUFB8, NDUFC1, NDUFC2, NDUFS1, NDUFV1, NDUFS5, NDUFS6, NDUFV2, SDHA, SDHB, NDUFA12, NDUFA11, NDUFS7
1	KEGG Pathway	hsa05010	Alzheimer's disease	-28.38	-25.50	19/171	COX7C, NDUFA2, NDUFA5, NDUFA10, NDUFB3, NDUFB4, NDUFB8, NDUFC1, NDUFC2, NDUFS1, NDUFV1, NDUFS5, NDUFS6, NDUFV2, SDHA, SDHB, NDUFA12, NDUFA11, NDUFS7
1	KEGG Pathway	ko05010	Alzheimer's disease	-28.38	-25.50	19/171	COX7C, NDUFA2, NDUFA5, NDUFA10, NDUFB3, NDUFB4, NDUFB8, NDUFC1, NDUFC2, NDUFS1, NDUFV1, NDUFS5, NDUFS6, NDUFV2, SDHA, SDHB, NDUFA12, NDUFA11, NDUFS7
1	GO Biological Processes	GO:0015980	energy derivation by oxidation of	-27.86	-24.99	21/278	COX7C, ND1, NDUFA2, NDUFA5, NDUFA10,

			organic compounds				NDUFB3, NDUFB4, NDUFB8, NDUFC1, NDUFC2, NDUFS1, NDUFV1, NDUFS5, NDUFS6, NDUFV2, SDHA, SDHB, CISD1, NDUFA12, NDUFA11, NDUFS7
1	WikiPathways	WP623	Oxidative phosphorylation	-27.75	-24.90	15/62	ND1, NDUFA2, NDUFA5, NDUFA10, NDUFB4, NDUFB8, NDUFC1, NDUFC2, NDUFS1, NDUFV1, NDUFS5, NDUFS6, NDUFV2, NDUFA11, NDUFS7
1	KEGG Pathway	hsa04723	Retrograde endocannabinoid signaling	-24.63	-21.79	17/167	ND1, NDUFA2, NDUFA5, NDUFA10, NDUFB3, NDUFB4, NDUFB8, NDUFC1, NDUFC2, NDUFS1, NDUFV1, NDUFS5, NDUFS6, NDUFV2, NDUFA12, NDUFA11, NDUFS7
1	GO Biological Processes	GO:0006091	generation of precursor metabolites and energy	-23.60	-20.78	22/524	COX7C, FECH, ND1, NDUFA2, NDUFA5, NDUFA10, NDUFB3, NDUFB4, NDUFB8, NDUFC1, NDUFC2, NDUFS1, NDUFV1, NDUFS5, NDUFS6, NDUFV2, SDHA, SDHB, CISD1, NDUFA12, NDUFA11, NDUFS7
1	GO Biological Processes	GO:0046034	ATP metabolic process	-23.26	-20.45	19/313	COX7C, ND1, NDUFA2, NDUFA5, NDUFA10, NDUFB3, NDUFB4, NDUFB8, NDUFC1, NDUFC2, NDUFS1, NDUFV1, NDUFS5, NDUFS6, NDUFV2, SDHA, NDUFA12, NDUFA11, NDUFS7
1	GO Biological Processes	GO:0007005	mitochondrion organization	-20.13	-17.33	20/548	GPX1, ND1, NDUFA2, NDUFA5, NDUFA10, NDUFB3, NDUFB4, NDUFB8, NDUFC1, NDUFC2, NDUFS1, NDUFV1, NDUFS5, NDUFS6, NDUFV2, COA4, NDUFA12,

							NDUFA11, NDUFS7, TIMM23
2	GO Biological Processes	GO:0009060	aerobic respiration	-6.18	-3.40	5/86	COX7C, ND1, SDHA, SDHB, NDUFS7
2	KEGG Pathway	hsa00020	Citrate cycle (TCA cycle)	-4.58	-1.83	3/30	PCK2, SDHA, SDHB
2	KEGG Pathway	ko00020	Citrate cycle (TCA cycle)	-4.58	-1.83	3/30	PCK2, SDHA, SDHB
2	GO Biological Processes	GO:0043648	dicarboxylic acid metabolic process	-3.06	-0.56	3/97	PCK2, SDHA, SDHB
3	Reactome Gene Sets	R-HSA-352230	Amino acid transport across the plasma membrane	-4.46	-1.72	3/33	SLC3A2, SLC7A5, SLC38A1
3	Reactome Gene Sets	R-HSA-425393	Transport of inorganic cations/anions and amino acids/oligopeptides	-4.25	-1.54	4/108	SLC3A2, SLC7A5, SLC4A7, SLC38A1
3	GO Biological Processes	GO:0015804	neutral amino acid transport	-4.08	-1.38	3/44	SLC3A2, SLC7A5, SLC38A1
3	KEGG Pathway	hsa04216	Ferroptosis	-4.02	-1.35	3/46	GPX4, SLC3A2, SLC38A1
3	GO Biological Processes	GO:1902475	L-alpha-amino acid transmembrane transport	-3.61	-0.96	3/63	SLC3A2, SLC7A5, SLC38A1
3	GO Biological Processes	GO:0015807	L-amino acid transport	-3.55	-0.94	3/66	SLC3A2, SLC7A5, SLC38A1
3	GO Biological Processes	GO:0003333	amino acid transmembrane transport	-3.08	-0.56	3/96	SLC3A2, SLC7A5, SLC38A1
3	GO Biological Processes	GO:0003018	vascular process in circulatory system	-2.90	-0.44	4/245	GPX1, YES1, SLC7A5, SLC38A1
3	Reactome Gene Sets	R-HSA-425407	SLC-mediated transmembrane transport	-2.86	-0.42	4/251	SLC3A2, SLC7A5, SLC4A7, SLC38A1
3	Reactome Gene Sets	R-HSA-202733	Cell surface interactions at the vascular wall	-2.63	-0.22	3/137	SLC3A2, YES1, SLC7A5
3	GO Biological Processes	GO:0006865	amino acid transport	-2.50	-0.11	3/153	SLC3A2, SLC7A5, SLC38A1
3	GO Biological Processes	GO:1905039	carboxylic acid transmembrane	-2.49	-0.11	3/154	SLC3A2, SLC7A5, SLC38A1

			transport				
3	GO Biological Processes	GO:1903825	organic acid transmembrane transport	-2.48	-0.11	3/155	SLC3A2, SLC7A5, SLC38A1
3	GO Biological Processes	GO:0098656	anion transmembrane transport	-2.40	-0.06	5/549	SLC3A2, SLC7A5, SLC4A7, SLC38A1, TIMM23
3	Reactome Gene Sets	R-HSA-71291	Metabolism of amino acids and derivatives	-2.24	0.00	4/374	ASNS, SLC3A2, SLC7A5, ADI1
3	GO Biological Processes	GO:0015711	organic anion transport	-2.23	0.00	4/377	SLC3A2, SLC7A5, SLC4A7, SLC38A1
3	GO Biological Processes	GO:0098657	import into cell	-2.01	0.00	3/230	SLC3A2, SLC7A5, SLC38A1
4	GO Biological Processes	GO:0006415	translational termination	-4.30	-1.57	4/105	ABCE1, MRPS16, MRPS33, MRPS10
4	Reactome Gene Sets	R-HSA-5368286	Mitochondrial translation initiation	-3.20	-0.65	3/87	MRPS16, MRPS33, MRPS10
4	Reactome Gene Sets	R-HSA-5389840	Mitochondrial translation elongation	-3.20	-0.65	3/87	MRPS16, MRPS33, MRPS10
4	Reactome Gene Sets	R-HSA-5419276	Mitochondrial translation termination	-3.20	-0.65	3/87	MRPS16, MRPS33, MRPS10
4	GO Biological Processes	GO:0070125	mitochondrial translational elongation	-3.17	-0.65	3/89	MRPS16, MRPS33, MRPS10
4	GO Biological Processes	GO:0070126	mitochondrial translational termination	-3.17	-0.65	3/89	MRPS16, MRPS33, MRPS10
4	Reactome Gene Sets	R-HSA-5368287	Mitochondrial translation	-3.12	-0.60	3/93	MRPS16, MRPS33, MRPS10
4	GO Biological Processes	GO:0043624	cellular protein complex disassembly	-3.06	-0.56	4/222	ABCE1, MRPS16, MRPS33, MRPS10
4	GO Biological Processes	GO:0032543	mitochondrial translation	-2.66	-0.23	3/134	MRPS16, MRPS33, MRPS10
4	GO Biological Processes	GO:0006414	translational elongation	-2.65	-0.23	3/135	MRPS16, MRPS33, MRPS10
4	GO Biological Processes	GO:0032984	protein-containing complex disassembly	-2.41	-0.06	4/334	ABCE1, MRPS16, MRPS33, MRPS10

4	GO Biological Processes	GO:0140053	mitochondrial gene expression	-2.41	-0.06	3/165	MRPS16, MRPS33, MRPS10
5	GO Biological Processes	GO:0045910	negative regulation of DNA recombination	-4.08	-1.38	3/44	H1-2, H1-10, NDFIP1
5	GO Biological Processes	GO:0045815	positive regulation of gene expression, epigenetic	-3.72	-1.06	3/58	H1-2, POLR2K, H1-10
5	GO Biological Processes	GO:0040029	regulation of gene expression, epigenetic	-3.19	-0.65	4/205	GPX1, H1-2, POLR2K, H1-10
5	GO Biological Processes	GO:0000018	regulation of DNA recombination	-2.98	-0.50	3/104	H1-2, H1-10, NDFIP1
5	GO Biological Processes	GO:0051053	negative regulation of DNA metabolic process	-2.76	-0.32	3/124	H1-2, H1-10, NDFIP1
5	GO Biological Processes	GO:0097549	chromatin organization involved in negative regulation of transcription	-2.59	-0.19	3/142	H1-2, HELLS, H1-10
5	GO Biological Processes	GO:0060968	regulation of gene silencing	-2.57	-0.18	3/144	H1-2, POLR2K, H1-10
5	GO Biological Processes	GO:0034401	chromatin organization involved in regulation of transcription	-2.49	-0.11	3/154	H1-2, HELLS, H1-10
5	GO Biological Processes	GO:0051052	regulation of DNA metabolic process	-2.32	0.00	4/355	H1-2, H1-10, GNL3, NDFIP1
5	GO Biological Processes	GO:0031497	chromatin assembly	-2.20	0.00	3/196	H1-2, HELLS, H1-10
5	GO Biological Processes	GO:0006333	chromatin assembly or disassembly	-2.05	0.00	3/221	H1-2, HELLS, H1-10
6	GO Biological Processes	GO:0009636	response to toxic substance	-4.02	-1.35	5/239	ASNS, FECH, GPX1, GPX4, MT1E
6	GO Biological Processes	GO:0006979	response to oxidative stress	-2.78	-0.34	5/447	GPX1, GPX4, ND1, NDUFB4, NDUFA12
6	GO Biological Processes	GO:0098754	detoxification	-2.62	-0.21	3/138	GPX1, GPX4, MT1E
6	GO Biological Processes	GO:0009416	response to light stimulus	-2.48	-0.11	4/320	ASNS, FECH, GPX1, POLD3
7	Reactome Gene Sets	R-HSA-1268020	Mitochondrial protein import	-3.59	-0.95	3/64	NDUFB8, COA4, TIMM23

7	Reactome Gene Sets	R-HSA-9609507	Protein localization	-2.42	-0.06	3/163	NDUFB8, COA4, TIMM23
8	Reactome Gene Sets	R-HSA-6782210	Gap-filling DNA repair synthesis and ligation in TC-NER	-3.57	-0.94	3/65	POLD2, POLR2K, POLD3
8	Reactome Gene Sets	R-HSA-6782135	Dual incision in TC-NER	-3.55	-0.94	3/66	POLD2, POLR2K, POLD3
8	KEGG Pathway	hsa00230	Purine metabolism	-3.46	-0.86	4/174	POLD2, POLR2K, PPAT, POLD3
8	KEGG Pathway	ko00230	Purine metabolism	-3.46	-0.86	4/174	POLD2, POLR2K, PPAT, POLD3
8	GO Biological Processes	GO:0006283	transcription-coupled nucleotide-excision repair	-3.42	-0.83	3/73	POLD2, POLR2K, POLD3
8	Reactome Gene Sets	R-HSA-6781827	Transcription-Coupled Nucleotide Excision Repair (TC-NER)	-3.32	-0.74	3/79	POLD2, POLR2K, POLD3
8	WikiPathways	WP4022	Pyrimidine metabolism	-3.22	-0.65	3/86	POLD2, POLR2K, POLD3
8	KEGG Pathway	hsa00240	Pyrimidine metabolism	-2.98	-0.50	3/104	POLD2, POLR2K, POLD3
8	KEGG Pathway	ko00240	Pyrimidine metabolism	-2.98	-0.50	3/104	POLD2, POLR2K, POLD3
8	GO Biological Processes	GO:0006289	nucleotide-excision repair	-2.91	-0.44	3/110	POLD2, POLR2K, POLD3
8	Reactome Gene Sets	R-HSA-5696398	Nucleotide Excision Repair	-2.89	-0.44	3/111	POLD2, POLR2K, POLD3
8	Reactome Gene Sets	R-HSA-157579	Telomere Maintenance	-2.87	-0.42	3/113	POLD2, POLR2K, POLD3
8	Reactome Gene Sets	R-HSA-73886	Chromosome Maintenance	-2.60	-0.19	3/141	POLD2, POLR2K, POLD3
8	GO Biological Processes	GO:0000723	telomere maintenance	-2.43	-0.07	3/161	POLD2, POLD3, GNL3
8	GO Biological Processes	GO:0032200	telomere organization	-2.34	0.00	3/174	POLD2, POLD3, GNL3
9	GO Biological Processes	GO:1901605	alpha-amino acid metabolic process	-2.22	0.00	3/192	ASNS, PPAT, ADI1
9	GO Biological Processes	GO:0048732	gland development	-2.09	0.00	4/414	ASNS, GPX1, PCK2, PPAT

Table S4. Top ten groups enriched pathways of Metascape analysis of up-regulated proteins in MO treatment. Pathways were enriched from GO Biological Processes, Reactome, KEGG, Canonical pathways and WikiPathways.

Group ID	Category	Term	Description	LogP	Log (q-value)	InTerm _InList	Genes
1	GO Biological Processes	GO:0006066	alcohol metabolic process	-14.02	-9.73	15/373	ACAT2, ALDH2, ALDH1A3, ALDH3A2, SCARB1, DHCR7, DHCR24, FDFT1, FDXR, G6PD, HMGCS1, IDI1, MSMO1, DHRS4, RDH11
1	Reactome Gene Sets	R-HSA-191273	Cholesterol biosynthesis	-12.84	-8.99	7/25	ACAT2, DHCR7, DHCR24, FDFT1, HMGCS1, IDI1, MSMO1
1	GO Biological Processes	GO:1901615	organic hydroxy compound metabolic process	-12.73	-8.99	16/555	ACAT2, ALDH2, ALDH1A3, ALDH3A2, SCARB1, DHCR7, DHCR24, FDFT1, FDXR, G6PD, HMGCS1, IDI1, LIPA, MSMO1, DHRS4, RDH11
1	GO Biological Processes	GO:0016125	sterol metabolic process	-12.69	-8.99	11/166	ACAT2, SCARB1, DHCR7, DHCR24, FDFT1, FDXR, G6PD, HMGCS1, IDI1, LIPA, MSMO1
1	GO Biological Processes	GO:0008203	cholesterol metabolic process	-11.59	-7.99	10/150	ACAT2, SCARB1, DHCR7, DHCR24, FDFT1, FDXR, G6PD, HMGCS1, IDI1, MSMO1
1	GO Biological Processes	GO:1902652	secondary alcohol metabolic process	-11.33	-7.81	10/159	ACAT2, SCARB1, DHCR7, DHCR24, FDFT1, FDXR, G6PD, HMGCS1, IDI1, MSMO1
1	GO Biological Processes	GO:0006695	cholesterol biosynthetic process	-11.10	-7.71	8/73	ACAT2, DHCR7, DHCR24, FDFT1, G6PD, HMGCS1, IDI1, MSMO1
1	GO Biological Processes	GO:1902653	secondary alcohol biosynthetic process	-11.10	-7.71	8/73	ACAT2, DHCR7, DHCR24, FDFT1, G6PD, HMGCS1, IDI1, MSMO1
1	GO Biological Processes	GO:0016126	sterol biosynthetic process	-10.82	-7.47	8/79	ACAT2, DHCR7, DHCR24, FDFT1, G6PD, HMGCS1, IDI1, MSMO1

1	GO Biological Processes	GO:0008202	steroid metabolic process	-10.75	-7.45	12/329	ACAT2, SCARB1, DHCR7, DHCR24, FDFT1, FDXR, G6PD, HMGCS1, IDI1, LIPA, MSMO1, DHRS4
1	GO Biological Processes	GO:0006694	steroid biosynthetic process	-10.54	-7.28	10/191	ACAT2, SCARB1, DHCR7, DHCR24, FDFT1, FDXR, G6PD, HMGCS1, IDI1, MSMO1
1	GO Biological Processes	GO:0044283	small molecule biosynthetic process	-10.17	-6.97	15/693	ACAT2, ALDH1A3, ALDOA, DHCR7, DHCR24, ENO2, FDFT1, FDXR, G6PD, HMGCS1, IDI1, MSMO1, UGDH, LGSN, COQ5
1	WikiPathways	WP197	Cholesterol Biosynthesis Pathway	-9.71	-6.56	5/15	DHCR7, FDFT1, HMGCS1, IDI1, MSMO1
1	KEGG Pathway	hsa00100	Steroid biosynthesis	-9.01	-5.91	5/20	DHCR7, DHCR24, FDFT1, LIPA, MSMO1
1	KEGG Pathway	ko00100	Steroid biosynthesis	-9.01	-5.91	5/20	DHCR7, DHCR24, FDFT1, LIPA, MSMO1
1	WikiPathways	WP4718	Cholesterol metabolism (includes both Bloch and Kandutsch-Russell pathways)	-8.84	-5.79	6/47	DHCR7, DHCR24, FDFT1, HMGCS1, IDI1, MSMO1
1	GO Biological Processes	GO:0008610	lipid biosynthetic process	-8.83	-5.79	14/729	ACAT2, ALDH1A3, ALDH3A2, SCARB1, DHCR7, DHCR24, FDFT1, FDXR, G6PD, HMGCS1, IDI1, PCYT2, MSMO1, SELENOI
1	Reactome Gene Sets	R-HSA-556833	Metabolism of lipids	-8.75	-5.73	14/740	ACAT2, ALDH3A2, DHCR7, DHCR24, FDFT1, FDXR, HMGCS1, IDI1, CTSA, PSAP, PCYT2, MSMO1, PLD3, SELENOI
1	Reactome Gene Sets	R-HSA-8957322	Metabolism of steroids	-8.55	-5.55	8/151	ACAT2, DHCR7, DHCR24, FDFT1, FDXR, HMGCS1, IDI1, MSMO1
1	GO Biological Processes	GO:0046165	alcohol biosynthetic process	-8.44	-5.50	8/156	ACAT2, DHCR7, DHCR24, FDFT1, G6PD, HMGCS1, IDI1, MSMO1
1	GO Biological Processes	GO:1901617	organic hydroxy compound biosynthetic process	-6.83	-3.98	8/251	ACAT2, DHCR7, DHCR24, FDFT1, G6PD, HMGCS1, IDI1, MSMO1

1	KEGG Pathway	M00095	C5 isoprenoid biosynthesis, mevalonate pathway	-5.82	-3.12	3/10	ACAT2, HMGCS1, IDI1
1	KEGG Pathway	hsa_M00095	C5 isoprenoid biosynthesis, mevalonate pathway	-5.82	-3.12	3/10	ACAT2, HMGCS1, IDI1
1	KEGG Pathway	M00101	Cholesterol biosynthesis, squalene 2,3-epoxide => cholesterol	-5.68	-3.01	3/11	DHCR7, DHCR24, MSMO1
1	KEGG Pathway	hsa_M00101	Cholesterol biosynthesis, squalene 2,3-epoxide => cholesterol	-5.68	-3.01	3/11	DHCR7, DHCR24, MSMO1
1	Reactome Gene Sets	R-HSA-2426168	Activation of gene expression by SREBF (SREBP)	-5.51	-2.88	4/42	DHCR7, FDFT1, HMGCS1, IDI1
1	GO Biological Processes	GO:0045540	regulation of cholesterol biosynthetic process	-5.24	-2.66	4/49	DHCR7, FDFT1, HMGCS1, IDI1
1	GO Biological Processes	GO:0106118	regulation of sterol biosynthetic process	-5.24	-2.66	4/49	DHCR7, FDFT1, HMGCS1, IDI1
1	Reactome Gene Sets	R-HSA-1655829	Regulation of cholesterol biosynthesis by SREBP (SREBF)	-5.04	-2.49	4/55	DHCR7, FDFT1, HMGCS1, IDI1
1	GO Biological Processes	GO:0090181	regulation of cholesterol metabolic process	-4.83	-2.31	4/62	DHCR7, FDFT1, HMGCS1, IDI1
1	KEGG Pathway	hsa00900	Terpenoid backbone biosynthesis	-4.72	-2.24	3/22	ACAT2, HMGCS1, IDI1
1	KEGG Pathway	ko00900	Terpenoid backbone biosynthesis	-4.72	-2.24	3/22	ACAT2, HMGCS1, IDI1
1	WikiPathways	WP1982	Sterol Regulatory Element-Binding Proteins (SREBP) signalling	-4.55	-2.16	4/73	SCARB1, FDFT1, HMGCS1, IDI1
1	GO Biological Processes	GO:1902930	regulation of alcohol biosynthetic process	-4.48	-2.10	4/76	DHCR7, FDFT1, HMGCS1, IDI1
1	GO Biological Processes	GO:0008299	isoprenoid biosynthetic process	-4.39	-2.03	3/28	ALDH1A3, HMGCS1, IDI1

1	GO Biological Processes	GO:0050810	regulation of steroid biosynthetic process	-4.16	-1.82	4/92	DHCR7, FDFT1, HMGCS1, IDI1
1	GO Biological Processes	GO:0046890	regulation of lipid biosynthetic process	-3.90	-1.64	5/203	SCARB1, DHCR7, FDFT1, HMGCS1, IDI1
1	GO Biological Processes	GO:0019218	regulation of steroid metabolic process	-3.68	-1.49	4/122	DHCR7, FDFT1, HMGCS1, IDI1
1	GO Biological Processes	GO:0019216	regulation of lipid metabolic process	-3.41	-1.28	6/402	SCARB1, DHCR7, FDFT1, HMGCS1, IDI1, PSAP
1	GO Biological Processes	GO:0006644	phospholipid metabolic process	-3.31	-1.19	6/420	SCARB1, FDFT1, HMGCS1, IDI1, PCYT2, SELENOI
1	GO Biological Processes	GO:0062012	regulation of small molecule metabolic process	-3.21	-1.12	6/438	AK4, DHCR7, NQO1, FDFT1, HMGCS1, IDI1
1	GO Biological Processes	GO:0008654	phospholipid biosynthetic process	-2.41	-0.45	4/269	HMGCS1, IDI1, PCYT2, SELENOI
2	WikiPathways	WP2884	NRF2 pathway	-10.16	-6.97	9/146	BLVRB, NQO1, FTH1, G6PD, GCLC, GCLM, HMOX1, SLC2A1, TXN
2	WikiPathways	WP2882	Nuclear Receptors Meta-Pathway	-8.33	-5.42	10/322	BLVRB, NQO1, FTH1, G6PD, GCLC, GCLM, HMOX1, MGST1, SLC2A1, TXN
2	GO Biological Processes	GO:0006979	response to oxidative stress	-6.99	-4.13	10/447	DHCR24, NQO1, G6PD, GCLC, GCLM, HMOX1, MGST1, TXN, FOSL1, TMEM161A
2	GO Biological Processes	GO:0062197	cellular response to chemical stress	-5.75	-3.07	8/349	NQO1, G6PD, HMOX1, MGST1, SLC2A1, TXN, ERRFI1, TMEM161A
2	GO Biological Processes	GO:0009991	response to extracellular stimulus	-4.76	-2.25	8/477	NQO1, G6PD, GCLM, HMGCS1, HMOX1, SLC2A1, FOSL1, GDF15
2	GO Biological Processes	GO:0042493	response to drug	-4.63	-2.19	7/359	AK4, NQO1, GCLM, HMGCS1, HMOX1, MGST1, FOSL1
2	GO Biological Processes	GO:0034599	cellular response to oxidative	-4.09	-1.77	6/301	NQO1, G6PD, HMOX1, MGST1, TXN,

			stress				TMEM161A
2	GO Biological Processes	GO:1901214	regulation of neuron death	-4.08	-1.77	6/302	CTSZ, DHCR24, NQO1, G6PD, GCLM, HMOX1
2	GO Biological Processes	GO:0031667	response to nutrient levels	-4.01	-1.72	7/451	NQO1, G6PD, GCLM, HMGCS1, HMOX1, SLC2A1, GDF15
2	GO Biological Processes	GO:0070997	neuron death	-3.78	-1.57	6/342	CTSZ, DHCR24, NQO1, G6PD, GCLM, HMOX1
2	GO Biological Processes	GO:0010035	response to inorganic substance	-3.52	-1.37	7/542	CA2, NQO1, G6PD, HMGCS1, HMOX1, TXN, FOSL1
2	GO Biological Processes	GO:0042542	response to hydrogen peroxide	-3.51	-1.36	4/135	NQO1, HMOX1, TXN, FOSL1
2	GO Biological Processes	GO:0007584	response to nutrient	-3.18	-1.10	4/165	NQO1, GCLM, HMGCS1, HMOX1
2	GO Biological Processes	GO:0043523	regulation of neuron apoptotic process	-2.90	-0.84	4/197	NQO1, G6PD, GCLM, HMOX1
2	GO Biological Processes	GO:0000302	response to reactive oxygen species	-2.69	-0.68	4/225	NQO1, HMOX1, TXN, FOSL1
2	GO Biological Processes	GO:0051402	neuron apoptotic process	-2.65	-0.65	4/230	NQO1, G6PD, GCLM, HMOX1
2	GO Biological Processes	GO:0097305	response to alcohol	-2.63	-0.63	4/234	NQO1, G6PD, HMGCS1, FOSL1
2	GO Biological Processes	GO:0043065	positive regulation of apoptotic process	-2.61	-0.62	6/574	ALDH1A3, CTSD, NQO1, HMOX1, FOSL1, ARHGEF7
2	GO Biological Processes	GO:0043068	positive regulation of programmed cell death	-2.55	-0.57	6/590	ALDH1A3, CTSD, NQO1, HMOX1, FOSL1, ARHGEF7
2	GO Biological Processes	GO:0010942	positive regulation of cell death	-2.34	-0.41	6/652	ALDH1A3, CTSD, NQO1, HMOX1, FOSL1, ARHGEF7
2	GO Biological Processes	GO:0010038	response to metal ion	-2.00	-0.11	4/352	CA2, NQO1, G6PD, HMOX1
3	KEGG Pathway	hsa00480	Glutathione metabolism	-8.47	-5.51	6/54	G6PD, GCLC, GCLM, MGST1, RRM2, RRM2B
3	KEGG Pathway	ko00480	Glutathione metabolism	-8.47	-5.51	6/54	G6PD, GCLC, GCLM, MGST1, RRM2, RRM2B
3	WikiPathways	WP3	Phytochemical activity on NRF2 transcriptional activation	-7.41	-4.51	4/15	NQO1, GCLC, GCLM, HMOX1

3	WikiPathways	WP3612	Photodynamic therapy-induced NFE2L2 (NRF2) survival signaling	-6.60	-3.78	4/23	NQO1, GCLC, GCLM, HMOX1
3	WikiPathways	WP4357	NRF2-ARE regulation	-6.60	-3.78	4/23	NQO1, GCLC, GCLM, HMOX1
3	WikiPathways	WP408	Oxidative Stress	-5.89	-3.17	4/34	NQO1, GCLC, HMOX1, MGST1
3	WikiPathways	WP4313	Ferroptosis	-5.60	-2.96	4/40	FTH1, GCLC, GCLM, HMOX1
3	KEGG Pathway	ko04216	Ferroptosis	-5.60	-2.96	4/40	FTH1, GCLC, GCLM, HMOX1
3	KEGG Pathway	hsa04216	Ferroptosis	-5.35	-2.73	4/46	FTH1, GCLC, GCLM, HMOX1
3	Reactome Gene Sets	R-HSA-211859	Biological oxidations	-4.82	-2.31	6/222	ALDH2, FDXR, GCLC, GCLM, MGST1, UGDH
3	WikiPathways	WP100	Glutathione metabolism	-4.66	-2.20	3/23	G6PD, GCLC, GCLM
3	Reactome Gene Sets	R-HSA-5579029	Metabolic disorders of biological oxidation enzymes	-4.14	-1.81	3/34	FDXR, GCLC, GCLM
3	Reactome Gene Sets	R-HSA-156590	Glutathione conjugation	-4.06	-1.76	3/36	GCLC, GCLM, MGST1
3	Reactome Gene Sets	R-HSA-156580	Phase II - Conjugation of compounds	-3.87	-1.61	4/109	GCLC, GCLM, MGST1, UGDH
3	GO Biological Processes	GO:0006790	sulfur compound metabolic process	-3.55	-1.38	6/378	G6PD, GCLC, GCLM, HMGCS1, MGST1, UGDH
3	WikiPathways	WP3940	One carbon metabolism and related pathways	-3.53	-1.37	3/54	GCLC, GCLM, PCYT2
3	Reactome Gene Sets	R-HSA-5668914	Diseases of metabolism	-3.53	-1.37	5/245	FDXR, GCLC, GCLM, GPC1, CTSA
3	GO Biological Processes	GO:0006749	glutathione metabolic process	-3.32	-1.20	3/64	G6PD, GCLC, GCLM
3	GO Biological Processes	GO:0009064	glutamine family amino acid metabolic process	-3.10	-1.03	3/76	GCLC, GCLM, LGSN
3	GO Biological Processes	GO:0044272	sulfur compound biosynthetic process	-2.93	-0.87	4/193	GCLC, GCLM, MGST1, UGDH
3	GO Biological Processes	GO:0006520	cellular amino acid metabolic process	-2.09	-0.18	4/332	NQO1, GCLC, GCLM, LGSN

4	GO Biological Processes	GO:0006720	isoprenoid metabolic process	-7.30	-4.41	7/143	ALDH1A3, ALDH3A2, FDFT1, GPC1, HMGCS1, IDI1, RDH11
4	GO Biological Processes	GO:0006721	terpenoid metabolic process	-6.29	-3.51	6/124	ALDH1A3, ALDH3A2, FDFT1, GPC1, HMGCS1, RDH11
4	Reactome Gene Sets	R-HSA-5365859	RA biosynthesis pathway	-4.72	-2.24	3/22	ALDH1A3, DHRS4, RDH11
4	Reactome Gene Sets	R-HSA-5362517	Signaling by Retinoic Acid	-3.83	-1.58	3/43	ALDH1A3, DHRS4, RDH11
4	GO Biological Processes	GO:0016101	diterpenoid metabolic process	-3.79	-1.57	4/114	ALDH1A3, ALDH3A2, GPC1, RDH11
4	GO Biological Processes	GO:0006081	cellular aldehyde metabolic process	-3.34	-1.21	3/63	ALDH1A3, ALDH3A2, RDH11
4	GO Biological Processes	GO:0001523	retinoid metabolic process	-2.66	-0.65	3/108	ALDH1A3, GPC1, RDH11
4	Reactome Gene Sets	R-HSA-9006931	Signaling by Nuclear Receptors	-2.25	-0.32	4/299	ALDH1A3, CTSD, DHRS4, RDH11
5	KEGG Pathway	hsa04142	Lysosome	-6.31	-3.52	6/123	CTSD, CTSZ, LIPA, CTSA, PSAP, SLC17A5
5	KEGG Pathway	ko04142	Lysosome	-6.31	-3.52	6/123	CTSD, CTSZ, LIPA, CTSA, PSAP, SLC17A5
5	GO Biological Processes	GO:0043299	leukocyte degranulation	-5.30	-2.69	9/537	ALDOA, CTSD, CTSZ, SERPINB1, FTH1, HMOX1, MGST1, CTSA, PSAP
5	GO Biological Processes	GO:0002275	myeloid cell activation involved in immune response	-5.22	-2.65	9/550	ALDOA, CTSD, CTSZ, SERPINB1, FTH1, HMOX1, MGST1, CTSA, PSAP
5	GO Biological Processes	GO:0002444	myeloid leukocyte mediated immunity	-5.20	-2.63	9/553	ALDOA, CTSD, CTSZ, SERPINB1, FTH1, HMOX1, MGST1, CTSA, PSAP
5	GO Biological Processes	GO:0045055	regulated exocytosis	-4.83	-2.31	10/780	ACTN4, ALDOA, CTSD, CTSZ, SERPINB1, FTH1, HMOX1, MGST1, CTSA, PSAP
5	Reactome Gene Sets	R-HSA-6798695	Neutrophil degranulation	-4.74	-2.24	8/480	ALDOA, CTSD, CTSZ, SERPINB1, FTH1, MGST1, CTSA, PSAP
5	GO Biological Processes	GO:0043312	neutrophil degranulation	-4.70	-2.23	8/486	ALDOA, CTSD, CTSZ, SERPINB1, FTH1, MGST1, CTSA, PSAP
5	GO Biological Processes	GO:0002283	neutrophil activation involved in	-4.68	-2.22	8/489	ALDOA, CTSD, CTSZ, SERPINB1, FTH1,

			immune response				MGST1, CTSA, PSAP
5	GO Biological Processes	GO:0002446	neutrophil mediated immunity	-4.61	-2.18	8/500	ALDOA, CTSD, CTSZ, SERPINB1, FTH1, MGST1, CTSA, PSAP
5	GO Biological Processes	GO:0042119	neutrophil activation	-4.61	-2.18	8/501	ALDOA, CTSD, CTSZ, SERPINB1, FTH1, MGST1, CTSA, PSAP
5	GO Biological Processes	GO:0002274	myeloid leukocyte activation	-4.58	-2.17	9/661	ALDOA, CTSD, CTSZ, SERPINB1, FTH1, HMOX1, MGST1, CTSA, PSAP
5	GO Biological Processes	GO:0036230	granulocyte activation	-4.58	-2.17	8/506	ALDOA, CTSD, CTSZ, SERPINB1, FTH1, MGST1, CTSA, PSAP
5	GO Biological Processes	GO:0002366	leukocyte activation involved in immune response	-4.30	-1.94	9/720	ALDOA, CTSD, CTSZ, SERPINB1, FTH1, HMOX1, MGST1, CTSA, PSAP
5	GO Biological Processes	GO:0002263	cell activation involved in immune response	-4.28	-1.93	9/724	ALDOA, CTSD, CTSZ, SERPINB1, FTH1, HMOX1, MGST1, CTSA, PSAP
5	Canonical Pathways	M3468	NABA ECM REGULATORS	-2.60	-0.61	4/238	CTSD, CTSZ, SERPINB1, CTSA
5	KEGG Pathway	hsa04210	Apoptosis	-2.36	-0.42	3/138	CTSD, CTSZ, LMNA
5	KEGG Pathway	ko04210	Apoptosis	-2.36	-0.42	3/138	CTSD, CTSZ, LMNA
5	Canonical Pathways	M5885	NABA MATRISOME ASSOCIATED	-2.05	-0.15	6/751	CTSD, CTSZ, SERPINB1, GPC1, CTSA, GDF15
6	KEGG Pathway	hsa00010	Glycolysis / Gluconeogenesis	-6.25	-3.50	5/67	ALDH2, ALDH1A3, ALDH3A2, ALDOA, ENO2
6	KEGG Pathway	ko00010	Glycolysis / Gluconeogenesis	-6.25	-3.50	5/67	ALDH2, ALDH1A3, ALDH3A2, ALDOA, ENO2
6	KEGG Pathway	hsa00280	Valine, leucine and isoleucine degradation	-5.28	-2.68	4/48	ACAT2, ALDH2, ALDH3A2, HMGCS1
6	KEGG Pathway	ko00280	Valine, leucine and isoleucine degradation	-5.28	-2.68	4/48	ACAT2, ALDH2, ALDH3A2, HMGCS1
6	KEGG Pathway	hsa00340	Histidine metabolism	-4.60	-2.18	3/24	ALDH2, ALDH1A3, ALDH3A2
6	KEGG Pathway	ko00340	Histidine metabolism	-4.60	-2.18	3/24	ALDH2, ALDH1A3, ALDH3A2

6	KEGG Pathway	hsa00053	Ascorbate and aldarate metabolism	-4.44	-2.07	3/27	ALDH2, ALDH3A2, UGDH
6	KEGG Pathway	ko00053	Ascorbate and aldarate metabolism	-4.44	-2.07	3/27	ALDH2, ALDH3A2, UGDH
6	KEGG Pathway	hsa00410	beta-Alanine metabolism	-4.26	-1.92	3/31	ALDH2, ALDH1A3, ALDH3A2
6	KEGG Pathway	ko00410	beta-Alanine metabolism	-4.26	-1.92	3/31	ALDH2, ALDH1A3, ALDH3A2
6	GO Biological Processes	GO:0034308	primary alcohol metabolic process	-4.12	-1.80	4/94	ALDH2, ALDH1A3, ALDH3A2, RDH11
6	KEGG Pathway	hsa00620	Pyruvate metabolism	-3.96	-1.68	3/39	ACAT2, ALDH2, ALDH3A2
6	KEGG Pathway	ko00620	Pyruvate metabolism	-3.96	-1.68	3/39	ACAT2, ALDH2, ALDH3A2
6	KEGG Pathway	hsa00380	Tryptophan metabolism	-3.92	-1.66	3/40	ACAT2, ALDH2, ALDH3A2
6	KEGG Pathway	ko00380	Tryptophan metabolism	-3.92	-1.66	3/40	ACAT2, ALDH2, ALDH3A2
6	WikiPathways	WP465	Tryptophan metabolism	-3.86	-1.61	3/42	ALDH2, ALDH3A2, DHCR24
6	KEGG Pathway	hsa00071	Fatty acid metabolism	-3.80	-1.57	3/44	ACAT2, ALDH2, ALDH3A2
6	KEGG Pathway	ko00071	Fatty acid degradation	-3.80	-1.57	3/44	ACAT2, ALDH2, ALDH3A2
6	KEGG Pathway	hsa00310	Lysine degradation	-3.42	-1.28	3/59	ACAT2, ALDH2, ALDH3A2
6	KEGG Pathway	ko00310	Lysine degradation	-3.42	-1.28	3/59	ACAT2, ALDH2, ALDH3A2
6	GO Biological Processes	GO:0044282	small molecule catabolic process	-3.23	-1.13	6/434	ACAT2, ALDH2, ALDH3A2, ALDOA, SCARB1, ENO2
6	GO Biological Processes	GO:0032787	monocarboxylic acid metabolic process	-2.37	-0.42	6/645	ACAT2, ALDH1A3, ALDH3A2, ALDOA, ENO2, MSMO1
7	Reactome Gene Sets	R-HSA-499943	Interconversion of nucleotide di- and triphosphates	-6.12	-3.38	4/30	AK4, RRM2, TXN, RRM2B
7	GO Biological Processes	GO:0090407	organophosphate biosynthetic process	-5.91	-3.18	10/587	AK4, ALDOA, G6PD, HMGCS1, IDI1, PCYT2, RRM2, NAMPT, RRM2B, SELENOI
7	GO Biological Processes	GO:0015949	nucleobase-containing small molecule interconversion	-4.44	-2.07	3/27	AK4, RRM2, RRM2B

7	Reactome Gene Sets	R-HSA-15869	Metabolism of nucleotides	-4.01	-1.72	4/100	AK4, RRM2, TXN, RRM2B
7	GO Biological Processes	GO:0055086	nucleobase-containing small molecule metabolic process	-3.95	-1.67	8/623	AK4, ALDOA, ENO2, HMGCS1, RRM2, UGDH, NAMPT, RRM2B
7	KEGG Pathway	hsanan01	drug metabolism	-3.75	-1.54	4/117	ALDH1A3, MGST1, RRM2, RRM2B
7	GO Biological Processes	GO:1901137	carbohydrate derivative biosynthetic process	-3.67	-1.48	8/684	AK4, ALDOA, G6PD, GPC1, RRM2, UGDH, RRM2B, STT3B
7	GO Biological Processes	GO:0009117	nucleotide metabolic process	-3.54	-1.37	7/538	AK4, ALDOA, ENO2, HMGCS1, RRM2, NAMPT, RRM2B
7	GO Biological Processes	GO:0006753	nucleoside phosphate metabolic process	-3.50	-1.35	7/547	AK4, ALDOA, ENO2, HMGCS1, RRM2, NAMPT, RRM2B
7	GO Biological Processes	GO:0009165	nucleotide biosynthetic process	-3.38	-1.25	5/264	AK4, ALDOA, RRM2, NAMPT, RRM2B
7	GO Biological Processes	GO:1901293	nucleoside phosphate biosynthetic process	-3.36	-1.23	5/267	AK4, ALDOA, RRM2, NAMPT, RRM2B
7	KEGG Pathway	hsa00230	Purine metabolism	-2.08	-0.18	3/174	AK4, RRM2, RRM2B
7	KEGG Pathway	ko00230	Purine metabolism	-2.08	-0.18	3/174	AK4, RRM2, RRM2B
8	WikiPathways	WP1946	Cori Cycle	-5.07	-2.51	3/17	ALDOA, G6PD, SLC2A1
8	GO Biological Processes	GO:0006734	NADH metabolic process	-3.80	-1.57	3/44	ALDOA, NQO1, ENO2
8	KEGG Pathway	hsa01200	Carbon metabolism	-3.79	-1.57	4/114	ACAT2, ALDOA, ENO2, G6PD
8	KEGG Pathway	ko01200	Carbon metabolism	-3.79	-1.57	4/114	ACAT2, ALDOA, ENO2, G6PD
8	WikiPathways	WP534	Glycolysis and Gluconeogenesis	-3.77	-1.56	3/45	ALDOA, ENO2, SLC2A1
8	KEGG Pathway	hsa04066	HIF-1 signaling pathway	-3.72	-1.52	4/119	ALDOA, ENO2, HMOX1, SLC2A1
8	GO Biological Processes	GO:0006091	generation of precursor metabolites and energy	-3.61	-1.43	7/524	AK4, ALDH2, ALDOA, ENO2, FDXR, G6PD, SDHAF2
8	GO Biological Processes	GO:0019674	NAD metabolic process	-3.61	-1.43	3/51	ALDOA, NQO1, ENO2
8	GO Biological Processes	GO:0005996	monosaccharide metabolic process	-3.32	-1.20	5/272	ALDOA, ENO2, G6PD, PGM5, SLC2A1

8	Canonical Pathways	M255	PID HIF1 TFPATHWAY	-3.28	-1.17	3/66	ALDOA, HMOX1, SLC2A1
8	GO Biological Processes	GO:0005975	carbohydrate metabolic process	-3.17	-1.10	7/620	ALDH2, ALDOA, ENO2, G6PD, PGM5, SLC2A1, UGDH
8	Reactome Gene Sets	R-HSA-71387	Metabolism of carbohydrates	-3.16	-1.08	5/295	ALDOA, ENO2, G6PD, GPC1, SLC2A1
8	WikiPathways	WP4018	Pathways in clear cell renal cell carcinoma	-2.93	-0.87	3/87	ALDOA, ENO2, SLC2A1
8	GO Biological Processes	GO:0006006	glucose metabolic process	-2.80	-0.76	4/210	ALDOA, ENO2, G6PD, PGM5
8	GO Biological Processes	GO:0016051	carbohydrate biosynthetic process	-2.80	-0.76	4/210	ALDOA, ENO2, G6PD, SLC2A1
8	GO Biological Processes	GO:0046364	monosaccharide biosynthetic process	-2.79	-0.76	3/97	ALDOA, ENO2, G6PD
8	GO Biological Processes	GO:0019318	hexose metabolic process	-2.52	-0.54	4/250	ALDOA, ENO2, G6PD, PGM5
8	GO Biological Processes	GO:0046031	ADP metabolic process	-2.51	-0.53	3/122	AK4, ALDOA, ENO2
8	GO Biological Processes	GO:0019693	ribose phosphate metabolic process	-2.42	-0.45	5/436	AK4, ALDOA, ENO2, G6PD, HMGCS1
8	GO Biological Processes	GO:0006165	nucleoside diphosphate phosphorylation	-2.41	-0.45	3/132	AK4, ALDOA, ENO2
8	GO Biological Processes	GO:0046939	nucleotide phosphorylation	-2.40	-0.45	3/133	AK4, ALDOA, ENO2
8	GO Biological Processes	GO:0009135	purine nucleoside diphosphate metabolic process	-2.38	-0.44	3/135	AK4, ALDOA, ENO2
8	GO Biological Processes	GO:0009179	purine ribonucleoside diphosphate metabolic process	-2.38	-0.44	3/135	AK4, ALDOA, ENO2
8	GO Biological Processes	GO:0009185	ribonucleoside diphosphate metabolic process	-2.36	-0.42	3/138	AK4, ALDOA, ENO2
8	GO Biological Processes	GO:0009132	nucleoside diphosphate metabolic process	-2.21	-0.29	3/156	AK4, ALDOA, ENO2
8	GO Biological Processes	GO:0046034	ATP metabolic process	-2.18	-0.26	4/313	AK4, ALDOA, ENO2, SDHAF2

9	GO Biological Processes	GO:0042445	hormone metabolic process	-4.87	-2.33	6/218	ALDH1A3, SCARB1, CTSZ, DHCR7, FDXR, RDH11
9	GO Biological Processes	GO:0010817	regulation of hormone levels	-4.53	-2.15	8/513	ALDH1A3, SCARB1, CTSZ, DHCR7, FDXR, SLC2A1, PHPT1, RDH11
9	GO Biological Processes	GO:0120178	steroid hormone biosynthetic process	-3.66	-1.48	3/49	SCARB1, DHCR7, FDXR
9	GO Biological Processes	GO:0034754	cellular hormone metabolic process	-3.50	-1.35	4/136	ALDH1A3, SCARB1, FDXR, RDH11
9	GO Biological Processes	GO:0042446	hormone biosynthetic process	-3.22	-1.13	3/69	SCARB1, DHCR7, FDXR
10	Canonical Pathways	M145	PID P53 DOWNSTREAM PATHWAY	-4.72	-2.24	5/137	CTSD, FDXR, SFN, GDF15, RRM2B
10	Reactome Gene Sets	R-HSA-5628897	TP53 Regulates Metabolic Genes	-4.25	-1.91	4/87	G6PD, SFN, TXN, RRM2B
10	WikiPathways	WP707	DNA Damage Response	-3.22	-1.13	3/69	SFN, RPA2, RRM2B
10	KEGG Pathway	hsa04115	p53 signaling pathway	-3.22	-1.13	3/69	SFN, RRM2, RRM2B
10	KEGG Pathway	ko04115	p53 signaling pathway	-3.22	-1.13	3/69	SFN, RRM2, RRM2B
10	WikiPathways	WP1530	miRNA Regulation of DNA Damage Response	-2.84	-0.79	3/93	SFN, RPA2, RRM2B
10	Reactome Gene Sets	R-HSA-3700989	Transcriptional Regulation by TP53	-2.75	-0.73	5/365	G6PD, SFN, RPA2, TXN, RRM2B

Table S5. Down-regulated proteins involved in the mitochondrial respiratory chain complex. Complex of this table represent mitochondrial respiratory chain complexes.

Gene names	Protein IDs	Protein names	P-value	Fold change	Complex
MT-ND1	P03886	NADH-ubiquinone oxidoreductase chain 1	0.023	0.593	I
NDUFS1	P28331	NADH-ubiquinone oxidoreductase 75 kDa subunit, mitochondrial	0.0009	0.854	I
NDUFS5	O43920	NADH dehydrogenase [ubiquinone] iron-sulfur protein 5	0.0117	0.714	I
NDUFS6	O75380	NADH dehydrogenase [ubiquinone] iron-sulfur protein 6, mitochondrial	0.0012	0.567	I
NDUFS7	O75251	NADH dehydrogenase [ubiquinone] iron-sulfur protein 7, mitochondrial	0.0004	0.618	I
NDUFV1	P49821	NADH dehydrogenase [ubiquinone] flavoprotein 1, mitochondrial	0.0001	0.713	I
NDUFV2	P19404	NADH dehydrogenase [ubiquinone] flavoprotein 2, mitochondrial	0.0038	0.805	I
NDUFA2	O43678	NADH dehydrogenase [ubiquinone] 1 alpha subcomplex subunit 2	0	0.703	I
NDUFA5	Q16718	NADH dehydrogenase [ubiquinone] 1 alpha subcomplex subunit 5	0.0002	0.761	I
NDUFA10	O95299	NADH dehydrogenase [ubiquinone] 1 alpha subcomplex subunit 10, mitochondrial	0.0251	0.644	I
NDUFA11	Q86Y39	NADH dehydrogenase [ubiquinone] 1 alpha subcomplex subunit 11	0.0006	0.666	I
NDUFA12	Q9UI09	NADH dehydrogenase [ubiquinone] 1 alpha subcomplex subunit 12	0.0019	0.77	I
NDUFB3	O43676	NADH dehydrogenase [ubiquinone] 1 beta subcomplex subunit 3	0.0037	0.644	I
NDUFB4	O95168	NADH dehydrogenase [ubiquinone] 1 beta subcomplex subunit 4	0.0019	0.673	I
NDUFB8	O95169	NADH dehydrogenase [ubiquinone] 1 beta subcomplex subunit 8, mitochondrial	0.0001	0.686	I
NDUFC1	O43677	NADH dehydrogenase [ubiquinone] 1 subunit C1, mitochondrial	0.0029	0.727	I
NDUFC2	O95298	NADH dehydrogenase [ubiquinone] 1 subunit C2	0.0009	0.781	I
SDHA	P31040	Succinate dehydrogenase [ubiquinone] flavoprotein subunit, mitochondrial	0.0035	0.77	II
SDHB	P21912	Succinate dehydrogenase [ubiquinone] iron-sulfur subunit, mitochondrial	0.0002	0.625	II
COX7C	P15954	Cytochrome c oxidase subunit 7C, mitochondrial	0.0146	0.794	IV
COA4	Q9NYJ1	Cytochrome c oxidase assembly factor 4 homolog, mitochondrial	0.0105	0.801	IV
